# Theoretical Model of the Island Effect in Flexible Electronics under Equal Biaxial Stretching

**DOI:** 10.1002/smll.202409632

**Published:** 2025-04-07

**Authors:** Zifei Ma, Tao Li, Xiaoyong Liu, Jun Wu, Haibo Luo, Yanchu Yang

**Affiliations:** ^1^ Aerospace Information Research Institute Chinese Academy of Sciences Beijing 100094 P. R. China; ^2^ School of Aeronautics and Astronautics University of Chinese Academy of Sciences Beijing 100040 P. R. China; ^3^ Hainan Research Institute Aerospace Information Research Institute Chinese Academy of Sciences Wenchang 571300 P. R. China

**Keywords:** equal biaxial stretching, flexible electronics, island effect, scaling law, strain concentration

## Abstract

Island‐bridge architecture represents a widely used structural design strategy in the field of stretchable inorganic electronics, where flexible serpentine interconnects function as bridges to accommodate most of the deformation, while the islands residing functional devices exhibit minimal deformation. The mismatch of Young's modulus between the elastic substrate and the rigid islands usually leads to stress concentration, which is named the island effect. This phenomenon can significantly increase the risk of interfacial damage, such as debonding and tearing failure near the island. In this work, the mechanical model of the island effect under equal biaxial stretching is developed based on theoretical analysis, simulation, and experimental measurement. The scaling law of the displacement and strain distributions on the substrate surface illustrates that the ratio of the island radius to the substrate length is the main controlling parameter of the island effect, proved by the experimental and numerical results. The criterion distinguishing whether the island effect problem is axisymmetric is derived from this. Further, the deformation field in periodic array structure is predicted based on the theoretical model, demonstrating its potential for application in flexible electronic devices.

## Graphical abstract

A mechanical model of the island effect under equal biaxial stretching is established through theoretical analysis, simulation, and experimental validation. The scaling law for displacement and strain distributions reveals that the ratio of island radius to substrate length is the key governing parameter. This model effectively predicts deformation fields in periodic arrays, highlighting its applicability in flexible electronic devices.

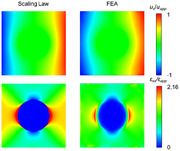

## 1 Introduction

Up to now, flexible and stretchable electronics that offer the performances of conventional devices^[^ ^1^, ^2^ ^]^ but with enhanced mechanical properties^[^ ^3^, ^4^ ^]^ have provided novel opportunities in various technical fields,^[^ ^5^, ^6^, ^7^, ^8^, ^9^, ^10^ ^]^ such as disease treatment and health monitoring,^[^ ^11^, ^12^, ^13^, ^14^, ^15^, ^16^, ^17^, ^18^, ^19^, ^20^, ^21^, ^22^, ^23^ ^]^ wireless communication,^[^ ^24^, ^25^, ^26^, ^27^, ^28^ ^]^ energy storage and harvest,^[^ ^29^, ^30^, ^31^, ^32^, ^33^, ^34^, ^35^, ^36^, ^37^, ^38^ ^]^ flexible display,^[^ ^36^ ^]^ soft robotics and micro‐fliers,^[^ ^39^, ^40^, ^41^, ^42^, ^43^ ^]^ and aerospace.^[^ ^44^, ^45^ ^]^

Flexible and stretchable electronics can be realized through various approaches.^[^ ^1^ ^]^ As for material‐based routes, novel functional materials with both superior mechanical and electrical performances, such as organic polymers,^[^ ^46^, ^47^ ^]^ carbon nanomaterials,^[^ ^48^, ^49^ ^]^ and self‐supporting functional ultra‐thin ultra‐flexible materials,^[^ ^50^, ^51^, ^52^, ^53^, ^54^, ^55^, ^56^ ^]^ have demonstrated significant potential in the field of flexible electronics. Meanwhile, a substantial proportion of flexible electronic systems adopting inorganic electronic components rely on innovative structural layouts to achieve high deformability.^[^ ^57^, ^58^, ^59^, ^60^, ^61^, ^62^, ^63^, ^64^, ^65^, ^66^ ^]^ Among various schemes of the flexible design, the island‐bridge structure^[^ ^67^, ^68^, ^69^ ^]^ is one of the most widely used design concepts, where non‐stretchable elements (i.e., islands) with deformable interconnects (i.e., bridges) are integrated onto the soft elastic substrate. This structural system can respond to sizable in‐plane load with the motion of planar serpentine bridges,^[^ ^58^, ^70^ ^]^ thereby realizing excellent stretchability.

However, due to the modulus mismatch of such rigid island‐soft substrate systems, a severe strain concentration will occur near the island boundary when the substrate is deformed (e.g., stretching). In this case, the material of the substrate can reach its mechanical limit locally even when the macroscopic load is relatively small, leading to substrate failure and reducing the system's stretchability and reliability. This so‐called “island effect” has been observed in previous literature.^[^ ^71^, ^72^, ^73^, ^74^, ^75^ ^]^ Wang et al. reported substantial localized stress at the rigid chip‐soft flexible printed circuit board (FPCB) substrate interface under bending. In this case, the soldering at the interface is prone to fracture, causing the chip detachment.^[^ ^76^ ^]^ Cantarella et al. presented an engineered elastomeric substrate with on‐top pillars, which localizes the strains on the substrate.^[^ ^77^ ^]^ Finite element analyses (FEA) simulations were performed to investigate the deformation behavior of the pillar‐substrate array subjected to stretching, bending, and twisting. Regarding the interpretation of the intrinsic mechanisms, Li et al. quantitively characterized the nonuniform deformation of a representative island‐substrate unit under uniaxial stretching.^[^ ^78^ ^]^ Except for such excellent research, the inner mechanism of the island effect and the impact of the rigid elements in more general scenarios (such as biaxial stretching, bending, and twisting) remain to be solved theoretically. Therefore, a theoretical model of this island effect with complex deformation mode is needed to predict the mechanical behavior of flexible‐rigid conformal structures.

This paper develops the scaling law for predicting the displacement and strain distribution of the island effect through theoretical, numerical, and experimental methods. According to the results of the theoretical model, the nonuniform deformation of the structural unit (consisting of a circular rigid island and a square elastic substrate) under equal biaxial stretching is solely influenced by the ratio of the island radius to the substrate length. Equal biaxial stretching experiments with digital image correlation (DIC) and finite element analyses (FEA) verify the applicability of the above conclusions. Prediction of the strain distribution in array configuration is provided as an example of its application prospects for the flexible‐rigid conformal structures in flexible electronics.

## 2 Mechanics Model of Island Effect

### 2.1 Geometric Model of Island Effect

**Figure 1:**
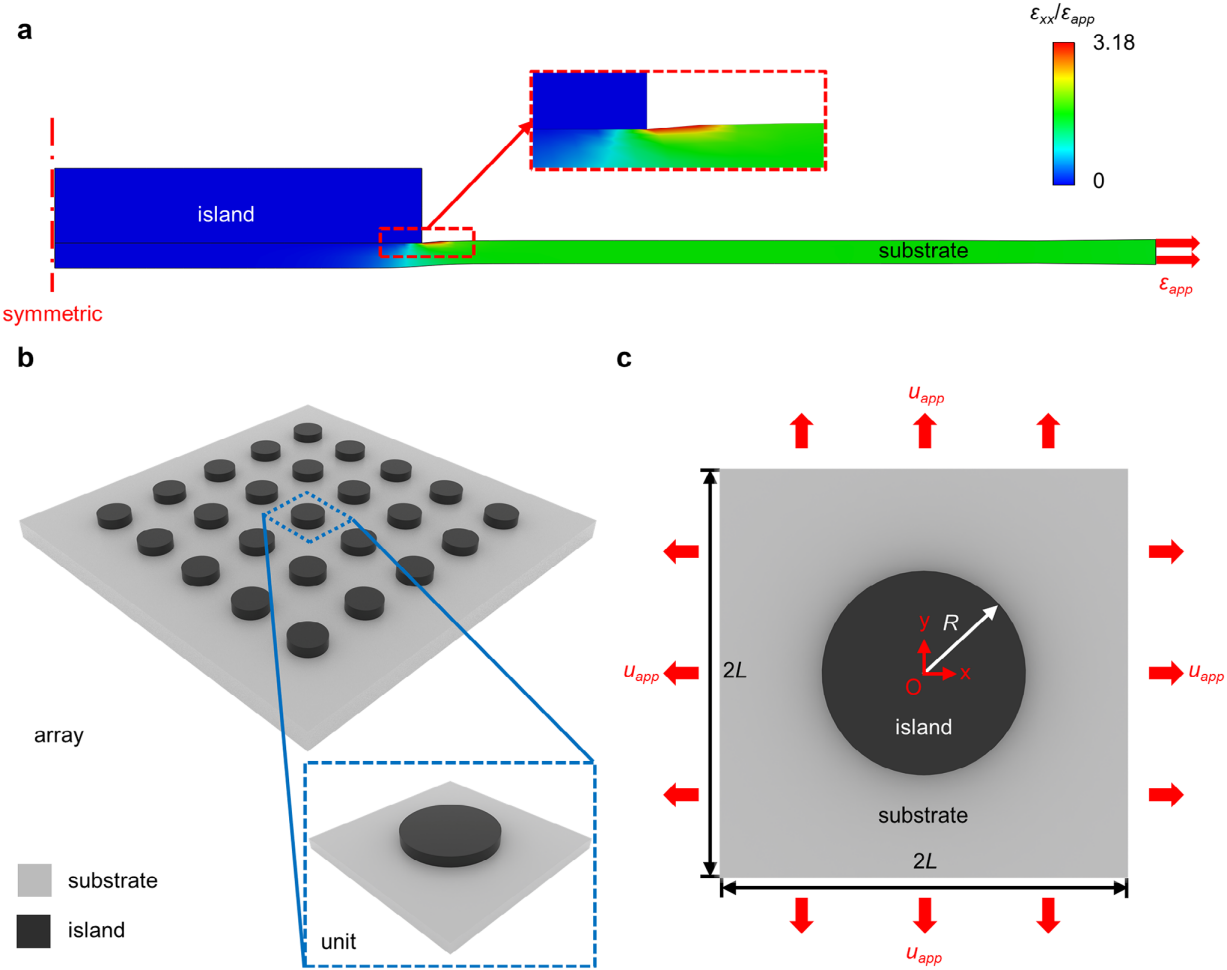
The geometric model of the island‐substrate structure with island effect. a) Contour plot of the normalized normal strain along the *x* direction in the island‐substrate structure (front view). b) A periodic array and a representative unit of the island‐substrate structure. c) Schematic diagram of the geometric model for the island effect.

**Figure** **1**a presents a schematic illustration of the island‐substrate system, consisting of a soft elastic square substrate and a bonding rigid circular island. When the substrate is stretched, the strain level at the interfacial boundary between the substrate and the island is significantly higher than the other regions, according to the finite element analysis (FEA) results. As the lower surface of the substrate remains free while the upper surface is constrained by the rigid island, the island‐substrate boundary experiences significant localized deformation during the tensile process to accommodate the uniform deformation at the bottom. Such an island effect may result in interfacial damage, such as debonding failure of the rigid island. Considering the hazards of strain concentration, establishing the mechanical model for predicting the island effect is essential and valuable.

Figure 1b illustrates a simplified 3D model of a periodic array in flexible electronic devices based on the design of island‐bridge structures. Since many contemporary stretchable electronics utilize suspended bridges detached from the substrate^[^ ^58^, ^79^, ^80^ ^]^ to enhance their stretchability, the mutual influence between the bridges and substrate is not significant (Figure S1, Supporting Information). Here, it is assumed that only the rigid islands are attached to the elastic substrate, and the effect of flexible interconnects (i.e., bridges) on the substrate can be disregarded. Therefore, the array is approximately composed of various island‐substrate units, as shown in Figure 1c. Since the non‐uniform deformation mainly occurs on the top surface of the substrate and its thickness is much smaller than the in‐plane sizes (length and width), the effect of out‐of‐plane dimension can be neglected (Figure S2, Supporting Information), and the model can be simplified as a 2D plane stress problem. Therefore, this study focuses on the in‐plane deformation of the substrate surface. The rigid island is supposed to be tightly attached to the elastic substrate without any relative displacement in the island region.

Based on the above assumptions, a 2D geometric model of the island‐substrate unit is established in Figure 1c. The circular island with radius *R* and elastic modulus *E_i_* is attached tightly to the square substrate with length/width 2*L* and elastic modulus *E_s_*. The geometric centers of the island and substrate coincide entirely. To simplify the theoretical model, the elastic substrate is stretched with equal biaxial displacement *u_app_*.

### 2.2 Scaling Law for Predicting the Displacement and Strain Distribution

#### 2.2.1 Key Variables that Determine the Displacement

Inspired by the geometrical symmetry of the island‐substrate unit, the polar coordinate system *Orθ* is set up to describe the deformation field of the substrate surface quantitatively. The geometric center of the unit is set as the pole, and the positive direction of the polar axis agrees with the *x*‐axis of the planar Cartesian coordinate system *Oxy* in Figure 1c. To simplify the model, all variables are dimensionless. The dimensionless displacement field of the substrate depends on five dimensionless parameters, as given by

(1) $$\frac{u_{r}}{u_{app}}=F_{1}\;\left(\varepsilon_{app},\frac{E_{s}}{E_{i}},\frac{R}{L},\frac{r}{L},\theta \right)$$

(2) $$\frac{u_{\theta }}{u_{app}}=F_{2}\;\left(\varepsilon_{app},\frac{E_{s}}{E_{i}},\frac{R}{L},\frac{r}{L},\theta \right)$$

Here, *ε_app_* = *u_app_*/*L* is the applied nominal strain, *E_s_*/*E_i_* is the Young's modulus ratio of the substrate to the island, *R*/*L* is the normalized island radius, *r*/*L* and *θ* are the dimensionless coordinates.

**Figure 2:**
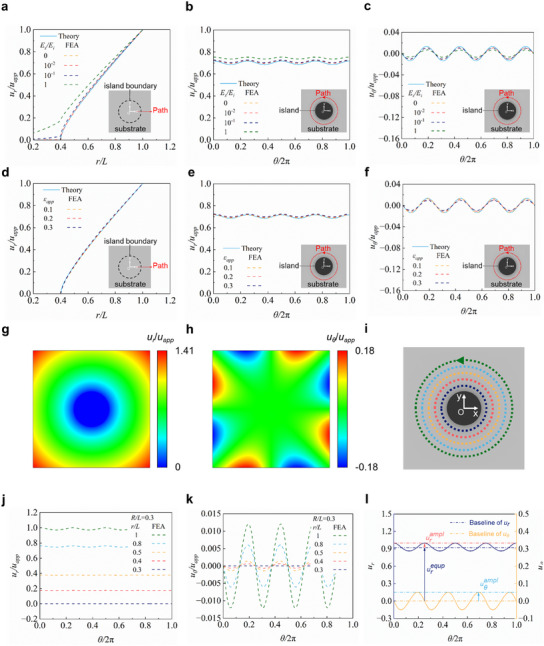
Establishment of the scaling law based on the displacement distribution. a) Distribution of normalized radial displacement (*u_r_*/*u_app_*) along radial direction (*θ* = 0) at the substrate surface with different levels of modulus. b) Distribution of *u_r_*/*u_app_* along a circular path where the normalized island radius (*r*/*L*) is equal to 0.8. c) Effect of the modulus ratio (*E_s_*/*E_i_*) on the normalized circumferential displacement (*u_θ_*/*u_app_*). d) Distribution of *u_r_*/*u_app_* along the radial direction under different applied strains (*ε_app_*). e) Distribution of *u_r_*/*u_app_* along the circular path where *r*/*L* = 0.8. f) Effect of *ε_app_* on *u_θ_*/*u_app_*. g) Contour plots of *u_r_*/*u_app_* for *R*/*L* = 0.3. h) Contour plots of *u_θ_*/*u_app_* for *R*/*L* = 0.3. i) Schematic diagram of circular paths with different levels of *r*/*L*. j) Distribution of *u_r_*/*u_app_* along the circumferential paths. k) Distribution of *u_θ_*/*u_app_* along the circumferential paths. l) Schematic diagram of displacement characteristic quantities (*u_r_^equp^*/*u_app_*, *u_r_^ampl^*/*u_app_*, and *u_θ_^ampl^*/*u_app_*).

Considering that the island effect is caused by the modulus mismatch between the elastic substrate and its on‐top island, the Young's modulus of the island is assumed to be much greater than the elastic modulus of the substrate (*E_s_*/*E_i_* ≈ 0). Therefore, the island can be regarded as a rigid part, and the deformation of the underneath substrate is negligible. **Figure** **2**a presents the distribution of radial displacement component *u_r_* along radial direction (*θ* = 0), with different modulus ratio *E_s_*/*E_i_*  = 0 (rigid island), 10^−2^, 10^−1^ and 1, controlling the constant values (*R*/*L* = 0.4 and *ε_app_* = 0.2). Note that the displacement results of the theory based on the scaling law agree well with the results of FEA based on ABAQUS simulations. And the selection for the constitutive model (linear elasticity or hyperelasticity) of the substrate material does not significantly affect the results of FEA, which could be negligible (Figure S3, Supporting Information). When the Young's modulus of the substrate is much smaller than that of the island (*E_s_*/*E_i_* ≤ 10^−1^), the deformation of the region under the island is almost equal to zero, and the differences among the displacement curves with different levels of *E_s_*/*E_i_* (≤10^−1^) are minimal. When the island and the substrate obtain the exact value of Young's modulus (*E_s_*/*E_i_* = 1), the distribution of *u_r_* is nearly bilinear. The region under the island shows a noticeable deformation, which is still smaller than the free surface deformation due to the influence of thickness. The distribution of displacement *u_r_* on the free surface with *E_s_*/*E_i_* = 1 exhibits minimal variation compared to those with *E_s_*/*E_i_* ≤ 10^−1^. The maximum discrepancy occurs at the boundary of the island with strain concentration. Figure 2b,c illustrates the relationship of displacement components (*u_r_*, *u_θ_*) and angle (*θ*) along the radial path (*r*/*L* = 0.8). The circumferential displacement *u_θ_* is far smaller than radial displacement *u_r_*, exhibiting minor sensitivity to Young's modulus ratio variations. Thus, the island can be assumed as a rigid part when the elastic modulus exceeds ten times that of the substrate, and the property of the substrate material shows negligible effect on the deformation pattern of the island effect (Figure S4, Supporting Information).

Figure 2d shows the distribution of radial displacement *u_r_* along radial direction (*θ* = 0) with different levels of applied strain (*ε_app_* = 0.1, 0.2, 0.3), controlling the constant values (*R*/*L* = 0.4 and *E_s_*/*E_i_* = 0). Figure 2e,f presents the distributions of displacement components (*u_r_* and *u_θ_*) along a radial path with *r/L* = 0.8, illustrating the normalized displacements (*u_r_*/*u_app_* and *u_θ_*/*u_app_*) are independent on the applied strain. Therefore, Equation (1) and (2) are rewritten as

(3) $$\frac{u_{r}}{u_{app}}=F_{1}\;\left(\frac{R}{L},\frac{r}{L},\theta \right)$$

(4) $$\frac{u_{\theta }}{u_{app}}=F_{2}\;\left(\frac{R}{L},\frac{r}{L},\theta \right)$$

Here, *R*/*L* is the key parameter of the island‐substrate system, controlling the deformation distribution.

#### 2.2.2 Framework of the Displacement Scaling Law

Figure 2g,h presents the contour plots of normalized displacement components, revealing that the island effect can induce non‐uniform deformation in the substrate. In this section, the impact of key parameters will be analyzed to derive the scaling law of the displacement distribution, and a quantitative expression for the strain field will be subsequently formulated through the differentiation method.

Considering the symmetry of displacement distribution in the circumferential direction, displacement data of the constant normalized island radius (*R*/*L* = 0.3) along five circular paths (*r*/*L* = 0.3, 0.4, 0.5, 0.8, and 1.0) have been extracted and analyzed, as shown in Figure 2i. In Figure 2j,k, the curves of the normalized displacement are close to those of the simple harmonic vibrations. Thus, the framework for the displacement scaling law can be established based on the trigonometric functions, as given by

(5) $$\frac{u_{r}}{u_{app}}=\left\{\begin{array}{cc} 0, & r\leq R\\ \frac{u_{r}^{equp}}{u_{app}}+\frac{u_{r}^{ampl}}{u_{app}}\cos4\theta , & r>R \end{array}\right.\;$$

(6) $$\frac{u_{\theta }}{u_{app}}=\left\{\begin{array}{cc} 0, & r\leq R\\ \frac{u_{\theta }^{equp}}{u_{app}}+\frac{u_{\theta }^{ampl}}{u_{app}}\sin\left(4\theta +\pi \right), & r>R \end{array}\right.\;$$

Here, *u_r_^equp^* and *u_θ_^equp^* denote the average values of radial and circumferential displacements, while *u_r_^ampl^* and *u_θ_^ampl^* denote the difference between the maximum and minimum values of displacement components on a circular path, as shown in Figure 2l. Due to the inherent symmetry and rotational periodicity, *u_θ_^equp^* always equals zero. The other three characteristic parameters (*u_r_^equp^/u_app_*, *u_r_^ampl^/u_app_* and *u_θ_^ampl^/u_app_*) are normalized based on dimensionless theory. According to Equations (5) and (6), the normalized displacement distribution is determined by the three parameters, which are controlled by normalized island radius (*R/L*) and the normalized radial coordinate (*r/L*).

#### 2.2.3 Characterization of Parameters in Scaling Law

Based on the FEA results, this section discusses the correlation between the normalized radial coordinate (*r*/*L*) and the normalized displacement characteristic parameters (*u_r_^equp^/u_app_*, *u_r_^ampl^/u_app_* and *u_θ_^ampl^/u_app_*). Then, the influence of the normalized island radius (*R*/*L*) is discussed, and the expression of the displacement scaling law is provided for predicting the displacement and strain distributions of the island effect.

**Figure 3:**
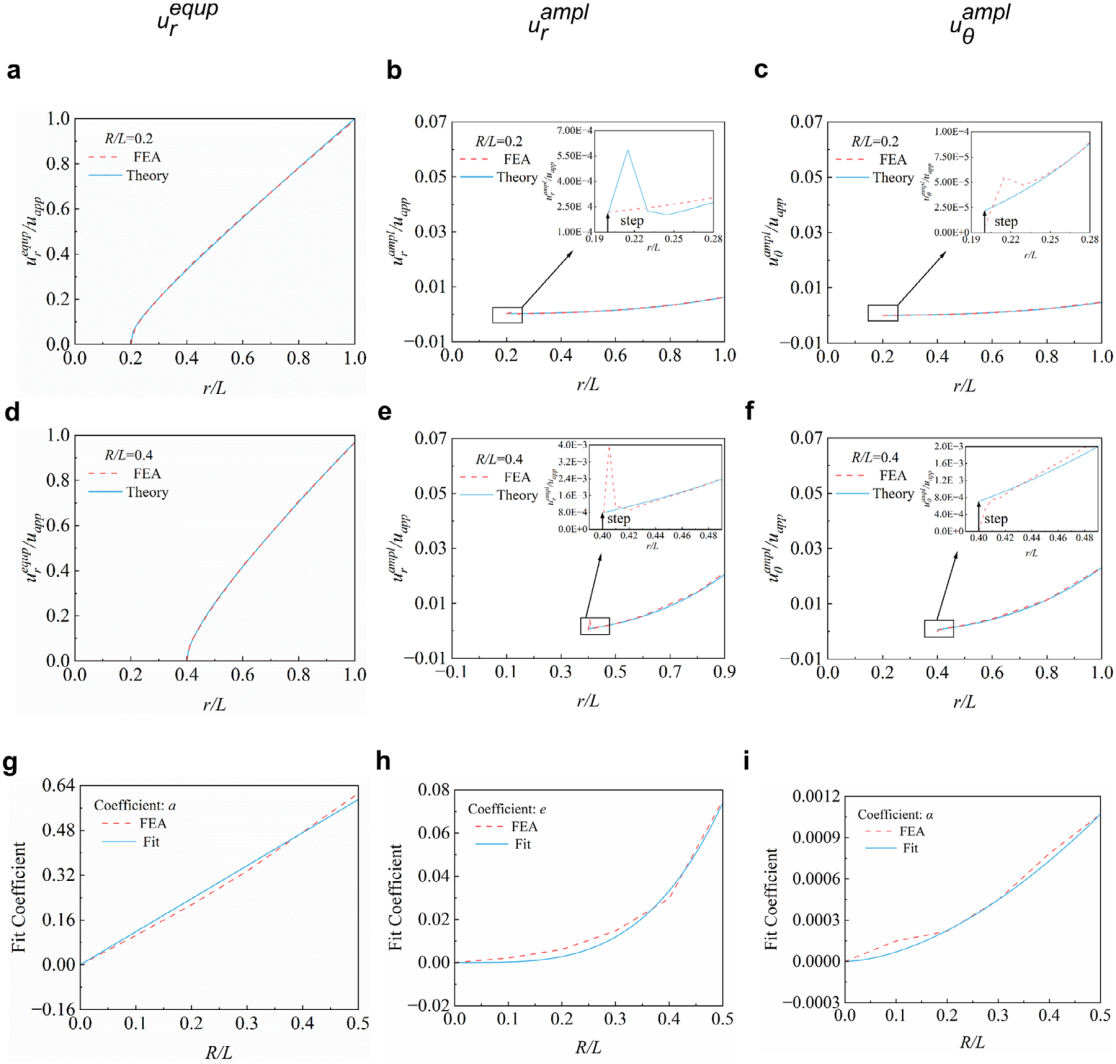
The framework of the scaling law with various coefficients. a) Distribution of *u_r_^equp^*/*u_app_* along the radial direction (*θ* = 0) for *R*/*L* = 0.2. b) Distribution of *u_r_^ampl^*/*u_app_* for *R*/*L* = 0.2. c) Distribution of *u_θ_^ampl^*/*u_app_* for *R*/*L* = 0.2. d–f) Distributions of the displacement components along radial direction for *R*/*L* = 0.4. g–i) Determination of the coefficients in the scaling law: *a, e*, and *α*.

**Figure** **3**a–f presents the relation curves between the normalized radial coordinate (*r*/*L*) and the normalized displacement characteristic parameters, with three different dimensionless island radii (*R*/*L* = 0.2, 0.4; for *R*/*L* = 0, Figure S5a–c, Supporting Information). The average radial displacement (*u_r_^equp^*) is much larger than the perturbation counterpart (*u_r_^ampl^*). The perturbation value of the radial and circumferential displacement (*u_r_^ampl^* and *u_θ_^ampl^*) grow faster when *r*/*L* increases. The average radial displacement (*u_r_^equp^*) experiences a rapid increase near the edge of the island before increasing linearly when moving away from the island. Furthermore, examining the displacement distribution for various scenarios provides a more comprehensive and in‐depth understanding of the deformation behavior and mechanisms underlying the island effect. Figure 3a,d shows that a larger island could induce a sharper increase of displacement (*u_r_^equp^/u_app_*) near the interfacial boundary (i.e., *r*/*L* = *R*/*L*), which signifies a higher degree of local strain concentration. As shown by Figure 3c,f, perturbation parameters (e.g., *u_θ_^ampl^/u_app_*) increase with the rise of *R*/*L*, indicating that the island size significantly influences the uniformity of the displacement distribution along the circumferential direction. Based on the above discussions, expressions of the displacement characteristic parameters can be obtained as

(7) $$\frac{u_{r}^{equp}}{u_{app}}=a{\left(\frac{r}{L}-\frac{R}{L}\right)}^{b}+\left(\frac{r}{L}-\frac{R}{L}\right)$$

(8) $$\frac{u_{r}^{ampl}}{u_{app}}=e\left({\left(\frac{r}{L}\right)}^{3}-{\left(\frac{R}{L}\right)}^{3}\right)+\alpha$$

(9) $$\frac{u_{\theta }^{ampl}}{u_{app}}=g\left({\left(\frac{r}{L}\right)}^{3}-{\left(\frac{R}{L}\right)}^{3}\right)+\beta$$

Here, *a, b, e, g, α, β* are all fitting coefficients determined by the dimensionless island radius (*R*/*L*). Through systematically extracting and summarizing additional FEA results (Figure 3g–i; Figure S5d–f, Supporting Information), all these above coefficients exhibit a positive correlation with the normalized island size. Thus, a set of empirical formulas can be developed via numerical fitting to illustrate the relationship between these coefficients and *R*/*L*, as given by

(10) $$a=1.179\left(\frac{R}{L}\right)$$

(11) $$b=1.224\left(\frac{R}{L}\right)$$

(12) $$e=0.8877{\left(\frac{R}{L}\right)}^{3.584}$$

(13) $$\alpha =3.484\times 10^{-3}{\left(\frac{R}{L}\right)}^{1.703}$$

(14) $$g=0.4383{\left(\frac{R}{L}\right)}^{2.976}$$

(15) $$\beta =4.586\times 10^{-2}{\left(\frac{R}{L}\right)}^{4.555}$$

By substituting Equations (7)–(9) into Equations (5) and (6), expressions of the displacement distribution are given by

(16) $$\begin{array}{c} \frac{u_{r}}{u_{app}}=\left\{\begin{array}{cc} 0, & r\leq R\\ a{\left(\frac{r}{L}-\frac{R}{L}\right)}^{b}+\left(\frac{r}{L}-\frac{R}{L}\right)+\left(e\left({\left(\frac{r}{L}\right)}^{3}-{\left(\frac{R}{L}\right)}^{3}\right)+\alpha \right)\cos4\theta , & r>R \end{array}\right. \end{array}$$

(17) $$\frac{u_{\theta }}{u_{app}}=\left\{\begin{array}{cc} 0, & r\leq R\\ \left(g\left({\left(\frac{r}{L}\right)}^{3}-{\left(\frac{R}{L}\right)}^{3}\right)+\beta \right)\sin\left(4\theta +\pi \right), & r>R \end{array}\right.$$

Thereby, the expressions of the strain distribution are given by

(18) $$\frac{\varepsilon_{rr}}{\varepsilon_{app}}=\left\{\begin{array}{cc} 0, & r\leq R\\ 1+ab{\left(\frac{r}{L}-\frac{R}{L}\right)}^{b-1}+3e{\left(\frac{r}{L}\right)}^{2}\cos4\theta , & r>R \end{array}\right.$$

(19) $$\frac{\varepsilon_{\theta \theta }}{\varepsilon_{app}}=\left\{\begin{array}{cc} 0, & r\leq R\\ \begin{array}{c} \frac{L}{r}\left(\frac{r}{L}-\frac{R}{L}+a{\left(\frac{r}{L}-\frac{R}{L}\right)}^{b}+\left(4g\left({\left(\frac{r}{L}\right)}^{3}-{\left(\frac{R}{L}\right)}^{3}\right)+e\left({\left(\frac{r}{L}\right)}^{3}-{\left(\frac{R}{L}\right)}^{3}\right)+\alpha -4\beta \right)\cos4\theta \right), \end{array} & r>R \end{array}\right.$$

(20) $$\frac{\varepsilon_{r\theta }}{\varepsilon_{app}}=\left\{\begin{array}{cc} 0, & r\leq R\\ \frac{L}{r}\left(g\left(2{\left(\frac{r}{L}\right)}^{3}+{\left(\frac{R}{L}\right)}^{3}\right)+4e\left({\left(\frac{r}{L}\right)}^{3}-{\left(\frac{R}{L}\right)}^{3}\right)+4\alpha -\beta \right)\sin\left(4\theta +\pi \right) & r>R \end{array}\right.$$

According to Equation (18), the coefficient *b* must be smaller than 1 to accurately characterize the strain concentration at the edge of the island (*r* = *R*). Based on Equation (11), *R*/*L* should be smaller than 0.81, which is the critical threshold *R_limit_*/*L*. When the dimensionless island radius exceeds this value, the above displacement and strain scaling law are no longer applicable.

When there is no island (*R*/*L* = 0), all coefficients are equal to zero, resulting in the linear distribution of radial displacement and the utterly uniform distribution of radial strain. According to Equation (18), coefficients *a* and *b* influence the uniformity of the radial strain distribution along the radial direction, indicating the existence of the rigid island promotes the strain concentration on the surface of the substrate. Coefficients *e* and *g* reflect the influence of the island on the displacement perturbation values (*u_r_^ampl^* and *u_θ_^ampl^*), respectively. When the values of *R/L* are equal to 0, 0.2, and 0.4, the corresponding values of *g* are 0, 3.646 × 10^−3^, and 2.868 × 10^−2^, and the maximum values of *u_θ_^ampl^/u_app_* exhibit a gradual increase from 0 to 4.763 × 10^−3^, and further to 2.320 × 10^−2^ (Figure 3c,f; Figure S5c, Supporting Information). Constants *α* and *β* denote the jump of displacement perturbation values at the edge of the island (Figure 3b,c,e,f).

In summary, the coefficients in our scaling law model provide a comprehensive characterization of the influence of rigid islands on the basal displacement field from multiple perspectives. Coefficient *b*, as the exponential term, quantifies the influence of the rigid island on the nonlinearity degree of the radial displacement distribution. Coefficient *a* affects the amplitude of the non‐uniform component in the radial displacement, while *e* and *g* denote the axisymmetric degree of the deformation field. In addition, constant terms *α* and *β* depict the abrupt jump of displacement perturbation values at the island boundary.

### 2.3 Simplification of the Scaling Law and Comparison with the Classical Theory

#### 2.3.1 Axisymmetric Simplification Criterion for the Scaling Law

According to the scaling law in the above section, the radial and circumferential displacements (*u_r_* and *u_θ_*) of larger islands exhibit a dependence on the angular coordinate *θ*. But for smaller islands, such influence can be ignored. Therefore, whether the deformation distribution of the island effect can be treated as an axisymmetric problem depends on the dimensionless island radius (*R*/*L*).

To simplify the scaling law efficiently and delineate between axisymmetric and non‐axisymmetric cases, the following axisymmetric simplification criterion is proposed, as given by

(21) $$\max\left(\frac{u_{r}^{ampl}}{u_{app}}\right)\leq 0.01\max\left(\frac{u_{r}^{equp}}{u_{app}}\right)$$

(22) $$\max\left(\frac{u_{\theta }^{ampl}}{u_{app}}\right)\leq 0.01\max\left(\frac{u_{r}^{equp}}{u_{app}}\right)$$

When Equations (21) and (22) are satisfied, the perturbation values (*u_r_^ampl^* and *u_θ_^ampl^*) are significantly smaller than the average value (*u_r_^equp^*). In this case, *θ* no longer influences the displacement distribution, which can be treated as an axisymmetric problem. Based on Equations (7) and (9), Equations (21) and (22) can be rewritten as

(23) $$\begin{array}{ccc} & & \max\left(e\left({\left(\frac{r}{L}\right)}^{3}-{\left(\frac{R}{L}\right)}^{3}\right)+\alpha \right)\leq \max\\ & & \;\left(0.01\left(a{\left(\frac{r}{L}-\frac{R}{L}\right)}^{b}+\left(\frac{r}{L}-\frac{R}{L}\right)\right)\right) \end{array}$$

(24) $$\begin{array}{ccc} & & \max\left(g\left({\left(\frac{r}{L}\right)}^{3}-{\left(\frac{R}{L}\right)}^{3}\right)+\beta \right)\leq \max\\ & & \;\left(0.01\left(a{\left(\frac{r}{L}-\frac{R}{L}\right)}^{b}+\left(\frac{r}{L}-\frac{R}{L}\right)\right)\right) \end{array}$$

According to Figure 3a–f, the displacement characteristic parameters (*u_r_^equp^*, *u* *_r_^ampl^* and *u_θ_^ampl^*) increase monotonically and the maximum values of both sides in the equations are obtained at *r = L*. Let *r/L* = 1, and the axisymmetric simplification criterion can be solved, as given by

(25) $$\frac{R}{L}\leq 0.2587$$

Thereby, the critical island radius *R_c_* satisfies

(26) $$R_{c}=0.2587L$$

Based on this criterion, the scaling law for predicting displacement and strain distribution is revised as

(27) $$\begin{array}{c} \frac{u_{r}}{u_{app}}=\left\{\begin{array}{cc} 0, & r\leq R\\ a{\left(\frac{r}{L}-\frac{R}{L}\right)}^{b}+\left(\frac{r}{L}-\frac{R}{L}\right), & R\leq R_{c}\;\&\;r>R\\ a{\left(\frac{r}{L}-\frac{R}{L}\right)}^{b}+\left(\frac{r}{L}-\frac{R}{L}\right)+\left(e\left({\left(\frac{r}{L}\right)}^{3}-{\left(\frac{R}{L}\right)}^{3}\right)+\alpha \right)\cos4\theta , & R>R_{c}\;\&\;r>R \end{array}\right. \end{array}$$

(28) $$\begin{array}{c} \frac{u_{\theta }}{u_{app}}=\left\{\begin{array}{cc} 0, & r\leq R\\ 0, & R\leq R_{c}\;\&\;r>R\\ \left(g\left({\left(\frac{r}{L}\right)}^{3}-{\left(\frac{R}{L}\right)}^{3}\right)+\beta \right)\sin\left(4\theta +\pi \right), & R>R_{c}\;\&\;r>R \end{array}\right. \end{array}$$

(29) $$\frac{\varepsilon_{rr}}{\varepsilon_{app}}=\left\{\begin{array}{cc} 0, & r\leq R\\ 1+ab{\left(\frac{r}{L}-\frac{R}{L}\right)}^{b-1}, & R\leq R_{c}\;\&\;r>R\\ 1+ab{\left(\frac{r}{L}-\frac{R}{L}\right)}^{b-1}+3e{\left(\frac{r}{L}\right)}^{2}\cos4\theta , & R>R_{c}\;\&\;r>R \end{array}\right.\;$$

(30) $$\frac{\varepsilon_{\theta \theta }}{\varepsilon_{app}}=\left\{\begin{array}{cc} 0, & r\leq R\\ \begin{array}{c} \frac{L}{r}\left(\frac{r}{L}-\frac{R}{L}+a{\left(\frac{r}{L}-\frac{R}{L}\right)}^{b}\right), \end{array} & R\leq R_{c}\;\&\;r>R\\ \begin{array}{c} \frac{L}{r}\left(\frac{r}{L}-\frac{R}{L}+a{\left(\frac{r}{L}-\frac{R}{L}\right)}^{b}+\left(4g\left({\left(\frac{r}{L}\right)}^{3}-{\left(\frac{R}{L}\right)}^{3}\right)+e\left({\left(\frac{r}{L}\right)}^{3}-{\left(\frac{R}{L}\right)}^{3}\right)+\alpha -4\beta \right)\cos4\theta \right), \end{array} & R>R_{c}\;\&\;r>R \end{array}\right.\;$$

(31) $$\frac{\varepsilon_{r\theta }}{\varepsilon_{app}}=\left\{\begin{array}{cc} 0, & r\leq R\\ 0, & R\leq R_{c}\;\&\;r>R\\ \frac{L}{r}\left(g\left(2{\left(\frac{r}{L}\right)}^{3}+{\left(\frac{R}{L}\right)}^{3}\right)+4e\left({\left(\frac{r}{L}\right)}^{3}-{\left(\frac{R}{L}\right)}^{3}\right)+4\alpha -\beta \right)\sin\left(4\theta +\pi \right) & R>R_{c}\;\&\;r>R \end{array}\right.\;$$

So far, the scaling law of the displacement/strain distribution on the substrate surface has been established, which describes the island effect well. When examining the radial displacement distribution (e.g., *u_r_*/*u_app_*), it also exhibits good consistency with previous literature^[^ ^78^ ^]^ (Figure S6, Supporting Information). Both can accurately predict the FEA results. This theoretical model can predict the deformation characteristics and mechanical behavior of flexible‐rigid conformal structures in flexible electronics. Moreover, provided that the following assumptions are satisfied, this scaling law model of the island effect is approximately valid:

1. The out‐of‐plane dimension (thickness) of the substrate is significantly smaller than its in‐plane sizes, allowing for the approximation as plane stress state.
2. The Young's modulus of the island is considerably greater than that of the substrate (*E_i_*/*E_s_* ≥10), so that it can be treated as a rigid body.
3. The dimensionless island radius *R*/*L* should be smaller than 0.81 (*R_limit_*/*L*, critical threshold).
4. The island‐substrate system is stretched with equal biaxial displacement.

#### 2.3.2 Comparison of Scaling Law with Classical Theory

When the size of the island is much smaller than that of the substrate, the displacement distribution on the substrate surface can be derived from classical elasticity theory (See Note S1, Supporting Information for details), as given by

(32) $$\frac{u_{r}}{u_{app}}=\left\{\begin{array}{cc} 0, & r\leq R\\ \frac{L}{r}\left(\frac{r^{2}-R^{2}}{L^{2}-R^{2}}\right), & r>R \end{array}\right.\;$$

(33) $$\frac{u_{\theta }}{u_{app}}\equiv 0$$

Here, the displacement field is axisymmetric and independent of angular coordinate *θ*.

Figure S7a–c (Supporting Information) provide the distribution curves of dimensionless radial displacement (*u_r_*/*u_app_*) along radial direction (*θ* = 0) with different normalized island radius (*R/L* = 0, 0.1, 0.2), based on the results of classical theory, scaling law, and FEA. When the axisymmetric simplification criterion is satisfied, the results of classical theory and scaling law are all in good agreement with FEA results.

Based on Equations (32) and (33), the strain distribution based on classical theory can be given by

(34) $$\frac{\varepsilon_{rr}}{\varepsilon_{app}}=\left\{\begin{array}{cc} 0, & r\leq R\\ \frac{L^{2}\left(r^{2}+R^{2}\right)}{r^{2}\left(L^{2}-R^{2}\right)}, & r>R \end{array}\right.\;$$

(35) $$\frac{\varepsilon_{\theta \theta }}{\varepsilon_{app}}=\left\{\begin{array}{cc} 0, & r\leq R\\ \frac{L^{2}\left(r^{2}-R^{2}\right)}{r^{2}\left(L^{2}-R^{2}\right)}, & r>R \end{array}\right.\;$$

(36) $$\frac{\varepsilon_{r\theta }}{\varepsilon_{app}}=0$$

**Figure 4:**
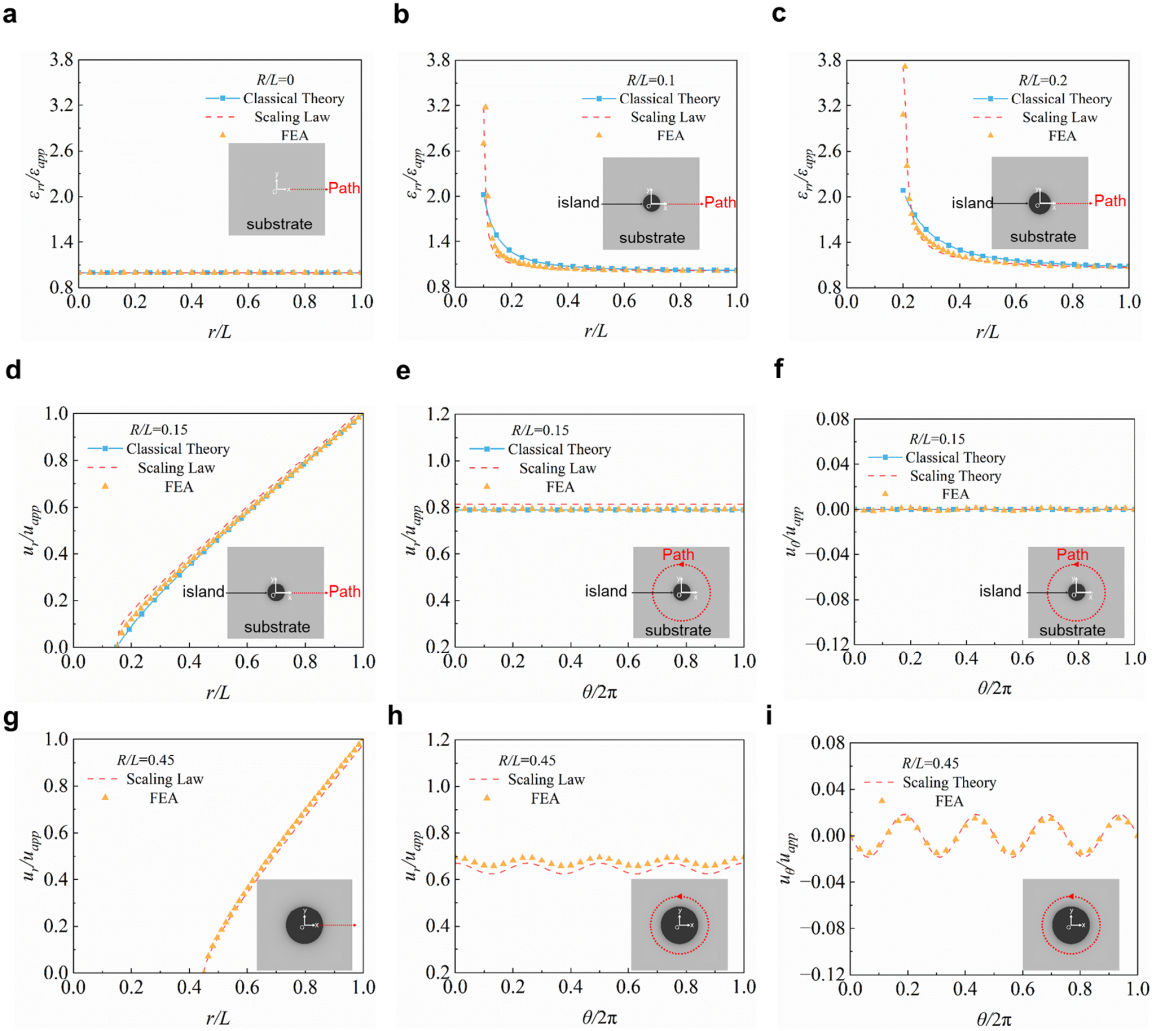
Distributions of the displacement and strain based on the scaling laws and the classical theory. a–c) Distribution of ε*_rr_*/*ε_app_* for *R*/*L* = 0, 0.1, and 0.2. d) Results of the classical theory, scaling law, and FEA about *u_r_*/*u_app_* along radial direction (*θ* = 0) for *R*/*L* = 0.15. e) Distribution of *u_r_*/*u_app_* along the circular path where *r*/*L* = 0.8. f) Distribution of *u_θ_*/*u_app_* along the circular path. g–i) Similar results for *R*/*L* = 0.45.

As shown in **Figure** **4**a–c, both the scaling law and the classical theory can effectively characterize the strain distribution in the cases of *R/L* = 0, 0.1, and 0.2. The contour plots of the normalized radial strain *ε_rr_*/*ε_app_* in the cases of *R*/*L* = 0.1 and 0.2 are shown in Figure S7d,e (Supporting Information). While the results of classical theory agree with the FEA results in general, the relatively significant differences near the island boundary demonstrate that the classical theory fails to adequately capture the strain concentration of the island effect.

#### 2.3.3 Predictive Capacity of the Scaling Law

To further validate the accuracy of the scaling law, Figure 4d–i presents the displacement fields in the cases of *R/L* = 0.15 and 0.45 based on the scaling law, compared with the FEA results.

Considering *R/L* = 0.15 satisfies the axisymmetric simplification criterion, additional curves based on classical theory are provided in Figure 4d–f. Figure 4d,g provides the variation of normalized radial displacement (*u_r_*/*u_app_*) along the radial direction (*θ* = 0) from the island boundary toward the substrate edge. Both the scaling law and classical theory can predict the displacement distribution well. Figure 4e,f demonstrates the influence of the angular coordinate on displacement by constraining the radial coordinate *r*/*L* to a constant value. When the island is small, *u_r_* is almost independent of *θ*. However, when the island is large (*R/L* = 0.45), the impact of *θ* on displacement is no longer negligible (Figure 4h,i).

## 3 Validation of the Scaling Law for the Island Effect

### 3.1 Assessment of Scaling Law Compliance with Ideal Boundary Conditions

Since the scaling law does not impose constraints on the unit boundaries, it is essential to ascertain whether boundary conditions under equal biaxial stretching can be satisfied. Considering the different contour shapes and constraints, internal and external boundary conditions are expressed in different forms. The inner boundary attached to the circular island is completely constrained without any displacement, and the corresponding boundary conditions are given by

(37) $$\left\{\begin{array}{c} \;{\left.\frac{u_{r}}{u_{app}}\right|}_{r=R}=0\\ \;{\left.\frac{u_{\theta }}{u_{app}}\right|}_{r=R}=0 \end{array}\right.$$

According to Equations (27) and (28), Equation (37) can be satisfied when the island is sufficiently small to meet the axisymmetric simplification criterion. When the island is large enough that the displacement field is non‐axisymmetric, the displacement distribution at the inner boundary based on the scaling law is given by

(38) $$\left\{\begin{array}{c} \;{\left.\frac{u_{r}}{u_{app}}\right|}_{r=R}=\alpha \cos4\theta \\ \;{\left.\frac{u_{\theta }}{u_{app}}\right|}_{r=R}=\beta \sin\left(4\theta +\pi \right) \end{array}\right.$$

According to Equation (13) and (15), when the normalized island radius (*R*/*L*) is smaller than the critical threshold (*R_limit_*/*L* = 0.81), the coefficient *α*, *β* is smaller than 2.433 × 10^−3^ and 1.756 × 10^−2^ respectively, which could be regarded as negligible small quantities. Therefore, the inner boundary conditions can be approximately satisfied based on the scaling law.

The outer boundaries of the substrate are stretched with equal biaxial displacements, and the ideal outer boundary conditions in a planar Cartesian coordinate system are given by

(39) $$\left\{\begin{array}{c} \;{\left.\frac{u_{x}}{u_{app}}\right|}_{x=L}=1\\ \;{\left.\frac{u_{y}}{u_{app}}\right|}_{y=L}=1\\ \;{\left.\frac{u_{x}}{u_{app}}\right|}_{x=-L}=-1\\ \;{\left.\frac{u_{y}}{u_{app}}\right|}_{y=-L}=-1 \end{array}\right.$$

Here, *u_x_* and *u_y_* denote the displacement components along the *x* and *y* directions, which can be calculated through coordinate transformation.

**Figure 5:**
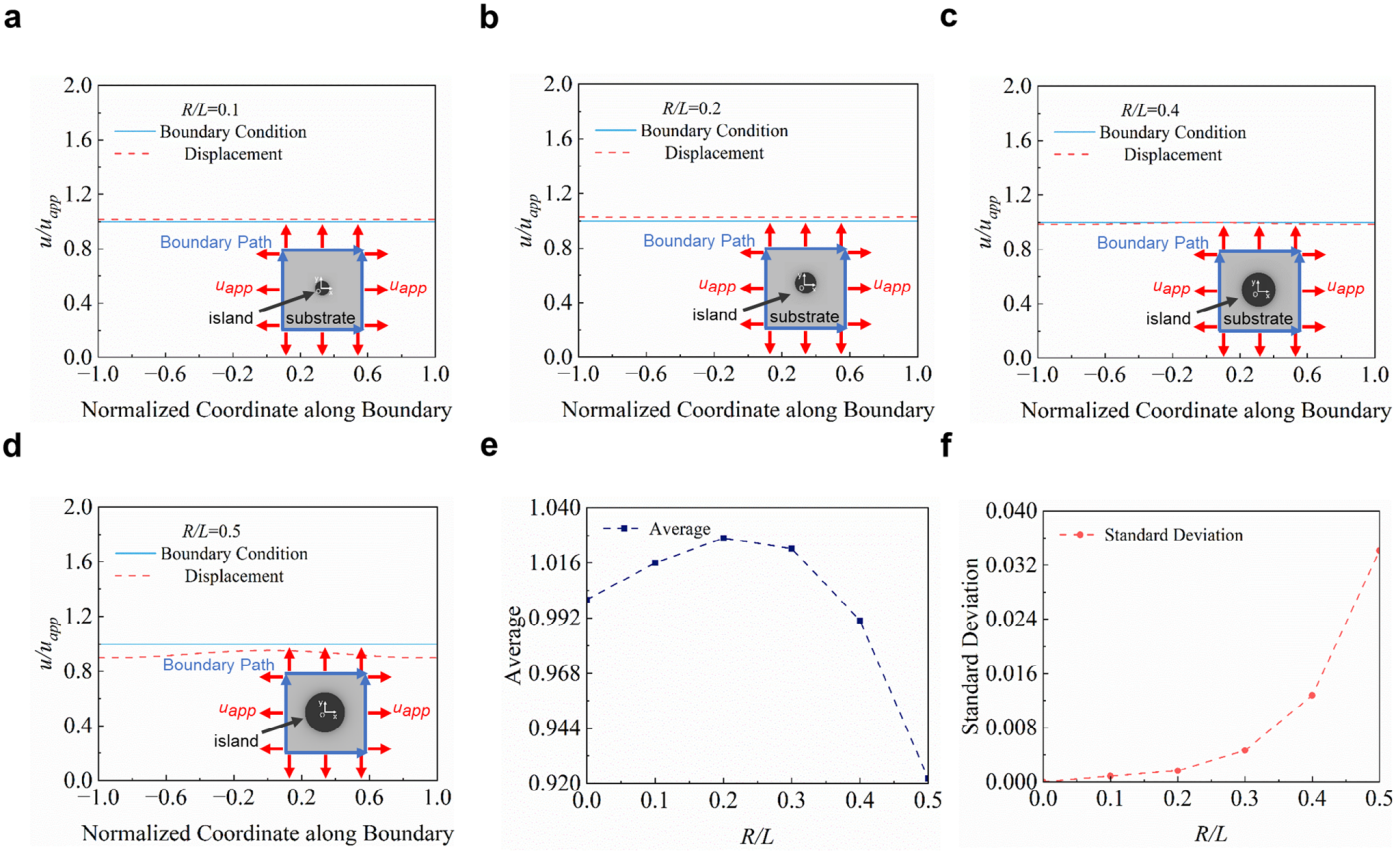
Verification of the scaling law about the ideal boundary conditions. a–d) Comparison of the displacement distribution at outer boundaries when *R*/*L* = 0.1, 0.2, 0.4, and 0.5. e,f) Mean value and standard deviation of the displacement distribution at the outer boundaries.

Due to the symmetry of the unit, the four boundary conditions can be satisfied when one of them is fulfilled. **Figure** **5**a–d present the displacement distribution at the edge derived from the scaling law and ideal boundary conditions for the island‐substrate system with various normalized island radii (*R/L* = 0.1, 0.2, 0.4, and 0.5; for *R*/*L* = 0 and 0.3, Figure S8a,b, Supporting Information). When the normalized island radius is smaller than 0.4, the displacement differences between the results of the scaling law and ideal boundary conditions are small and negligible. Only when the island is sufficiently large (e.g., *R/L* = 0.5) a deviation (<10%) can be noticed. According to Saint‐Venant's principle, the region far away from the island is hard to be influenced by the island, and the non‐uniform deformation occurs mainly at the edge of the island. The average displacement at the outer boundary of the substrate remains within 3% of the ideal boundary condition when the normalized island radius is smaller than 0.4 (Figure 5e), and the standard deviation of displacement at the boundary does not exceed 2% of the average value (Figure 5f).

In summary, the scaling law can approximately satisfy the ideal boundary conditions.

### 3.2 Experimental Verification of the Scaling Law

In this section, equal biaxial tensile tests with digital image correlation (DIC) are carried out to quantitatively measure the displacement and strain distribution on the substrate's top surface to validate the scaling law.

**Figure 6:**
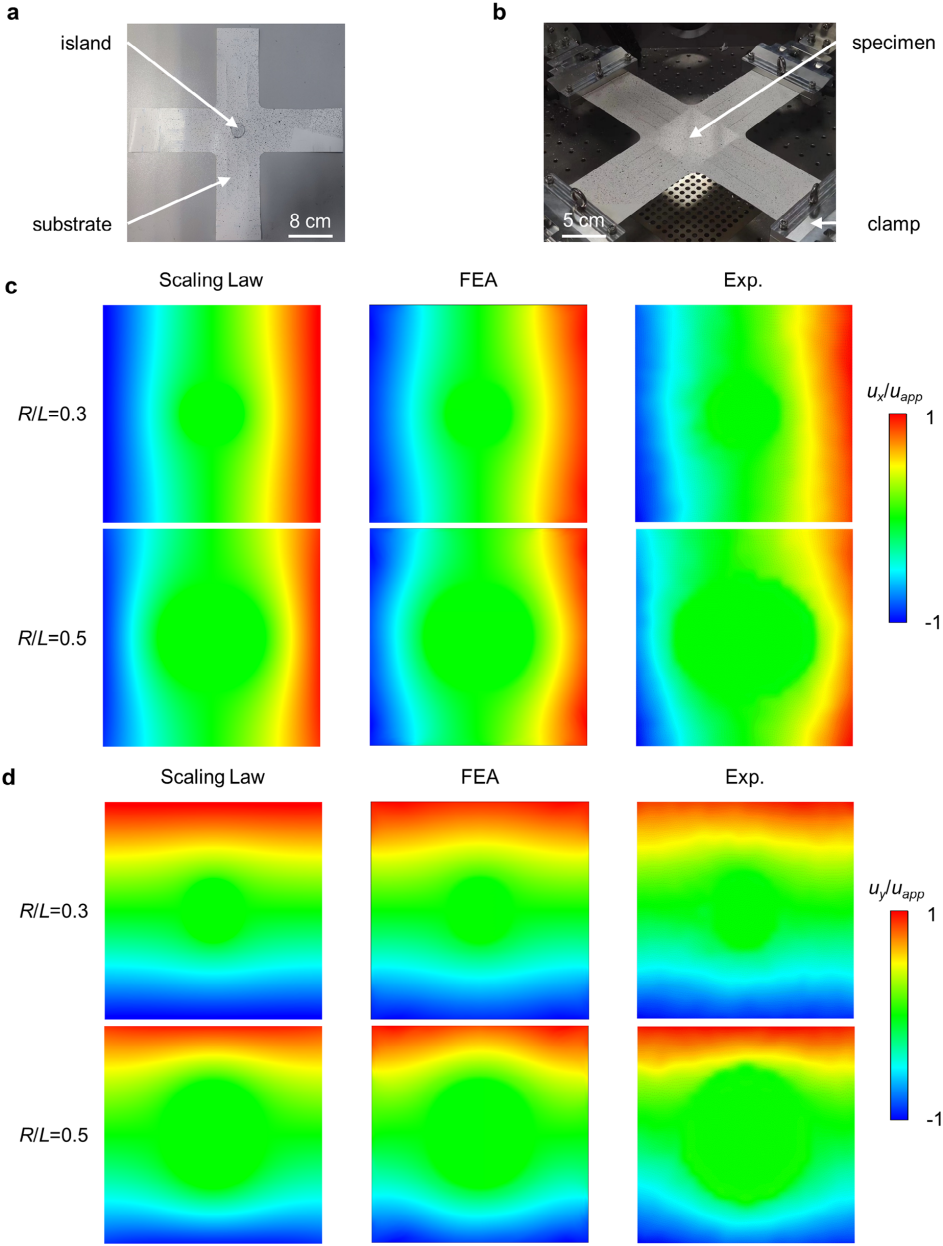
Experimental verification of scaling law for the island effect under small deformation. a,b) Optical image of the specimen and the experiment setup. c,d) Contour plots of normalized displacement along the *x* and *y* direction in the central test zone of the substrate based on the results of scaling law, FEA, and experiments. Scale bars, 8 cm in a) and 5 cm in b).

**Figure** **6**a,b presents the cross specimen fabricated by a customed textile material and the biaxial mechanical testing machine (Care Measurement & Control, China). Such textile material is characterized by relatively low in‐plane elongation (≤5%) and excellent bending flexibility, which can serve as the substrate for conformal devices on aerostats or smart fabrics. Figure 6c,d presents the contour plots of normalized displacement components along the *x*‐direction (*u_x_*/*u_app_*) and *y*‐direction (*u_y_*/*u_app_*). Note that the results of the experiments and FEA exhibit the central test region of the cross specimen, since the ideal biaxial stretching test is hard to be realized by the machine. The applied displacement *u_app_* is calculated based on the central test region rather than the side of the cross specimen. The average nominal strain in the central test region is ≈2%. The results of the scaling law are based on the 2D ideal geometric model of the unit, consisting of a circular rigid island attached to an elastic square substrate. When the normalized island radius (*R/L*) is larger, the inhomogeneity of the deformation induced by the rigid island is enhanced.

**Figure 7:**
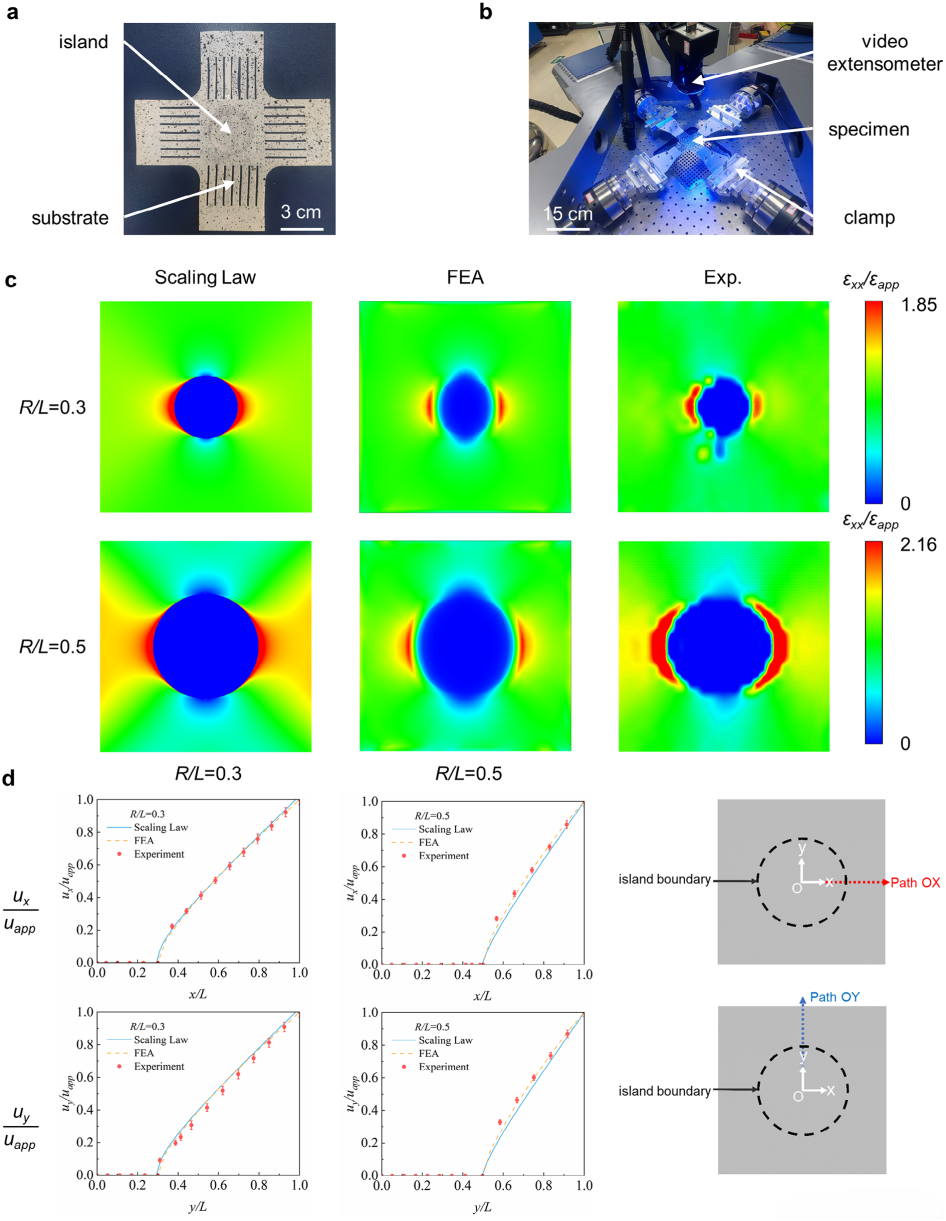
Experimental verification of scaling law for the island effect under large deformation. a,b) Optical image of the specimen and the experiment setup. c) Contour plots of normalized normal strain along the *x* direction for *R*/*L* = 0.3 and 0.5. d) Normalized displacement distribution (*u_x_*/*u_app_* and *u_y_*/*u_app_*) along the radial path for *R*/*L* = 0.3 and 0.5, based on the results of scaling law, FEA and experiments. Scale bars, 3 cm in a) and 15 cm in b).

To verify the accuracy of the scaling law for the island‐substrate system with large deformation, more flexible substrates fabricated by silicone (Ecoflex 0050A/B, Smooth‐On, USA) are selected for equal biaxial tensile tests, with the displacement and strain distribution measured (**Figure** **7**a). Such silicone material obtains significant large elongation (≥50%), which is widely used in the flexible electronics and wearable devices for health monitoring or disease treatment. Note that the deformation distribution of the textile and silicone materials under equal biaxial stretching (*ε_app_* ≈2%) shows small differences based on the experimental results (Figure S4, Supporting Information). The white specimen with black spots is stretched with large deformation by the experimental machine (Figure 7b). Due to the reduction in specimen size, adapters are mounted onto the clamps.

Figure 7c presents the distribution of the normalized normal strain component along the *x*‐direction (*ε_xx_*/*ε_app_*). Since the rigid island remains firmly adhered to the elastic substrate after stretching (Figure S9, Supporting Information), the results from digital image correlation are free from interfacial failure and can accurately reveal the influence of the rigid island on the substrate's deformation field. Cyclic loading test (Figure S10 and Movie S1, Supporting Information) further validates the durability of the island‐substrate structure and the stability concerning the normalized deformation pattern of the island effect. Due to the limited resolution of the DIC device and the insufficient density of the speckle, there are some deviations among the results of the scaling law, FEA, and experiments at the edge of the island with strain concentration. Overall, the results are generally in good agreement. When the normalized island radius (*R/L*) increases from 0.3 to 0.5, the maximum of the normalized strain rises from 1.85 to 2.16, correspondingly. This suggests that the enhancement of the island size will significantly promote the strain concentration of the island effect. Figure 7d presents the distribution of the normalized displacement components along the *x*‐direction (*u_x_*/*u_app_*) and *y*‐direction (*u_y_*/*u_app_*), with different normalized island radii (*R/L* = 0.3, 0.5). The displacement curves exhibit a sharp increase near the edge of the island, indicating the local strain concentration observed in Figure 7c. In addition, the errors of the experimental displacement data are generally within an acceptable range. These errors are relatively smaller at the beginning of the non‐zero segment of the curve, while being comparatively larger along the *x*‐direction. From the perspective of the experimental design and setup, the displacement near the island is relatively small. Furthermore, the outer boundary of the substrate (i.e., *r*/*L* = 1) is adjacent to the loading arm of the cross‐shaped specimen, which deviates from the boundary of equal biaxial state. Additionally, the speckles in this region are relatively sparse and more susceptible to distortion and failure during tensile process, reducing the accuracy of the value. In summary, there is no fundamental discrepancy between the simulation and experimental results. Through rigorous examination of the simulation modeling and comparison with existing theoretical framework,^[^ ^78^ ^]^ the accuracy and reliability of theoretical model here can be validated (Figure S6, Supporting Information). The observed errors and their impacts are acceptable and do not compromise the mutual validation among our theoretical model, FEA simulation and the experiment.

### 3.3 Island Effect of Periodic Array Structures

For the applications of flexible electronics and devices, it is common to encounter a periodic array of islands distributed on the substrate rather than a single island. In this section, the deformation of the elastic flexible substrate with a periodic island array is analyzed to assess the effectiveness of the scaling law for the applications of multiple islands.

The side length of the square substrate is 2*L* and the area is *S_s_*. On the substrate, the number of rigid islands with radius *R* is *n*, and the total area of the islands is *S_i_*. Thus, the area coverage ratio can be given by

(40) $$\eta =\frac{S_{i}}{S_{s}}=\frac{n\pi }{4}\;{\left(\frac{R}{L}\right)}^{2}$$

According to Equations ((29), (30), (31)), *R/L* is the only key parameter influencing the strain distribution. Consequently, the island effect does not obtain any scale‐dependent characteristics at the macroscopic scale, and the island‐substrate structures with identical *R/L* show similar normalized strain distributions. Analysis and testing of various island shapes,^[^ ^73^, ^81^ ^]^ including irregular island^[^ ^82^, ^83^, ^84^ ^]^ (Figure S11, Supporting Information), further confirm the dominant influence of the area coverage ratio (*η*) on the global strain concentration level. Note that all these shapes are designed with filleted corner and without sharp corner for reducing strain concentration. The observed differences may be attributed to the curvature of the island's shape. Furthermore, suppose the area coverage ratio *η* remains constant. In that case, the degree of strain concentration caused by an array of small rigid islands is equivalent to that caused by a large single rigid island. Thus, the equivalent radius of the array structure can be defined as

(41) $$R_{eq}=R\sqrt{n}$$

In ideal conditions, the arrays with the same equivalent radius obtain the same degree of strain concentration and similar deformation distribution.

**Figure 8:**
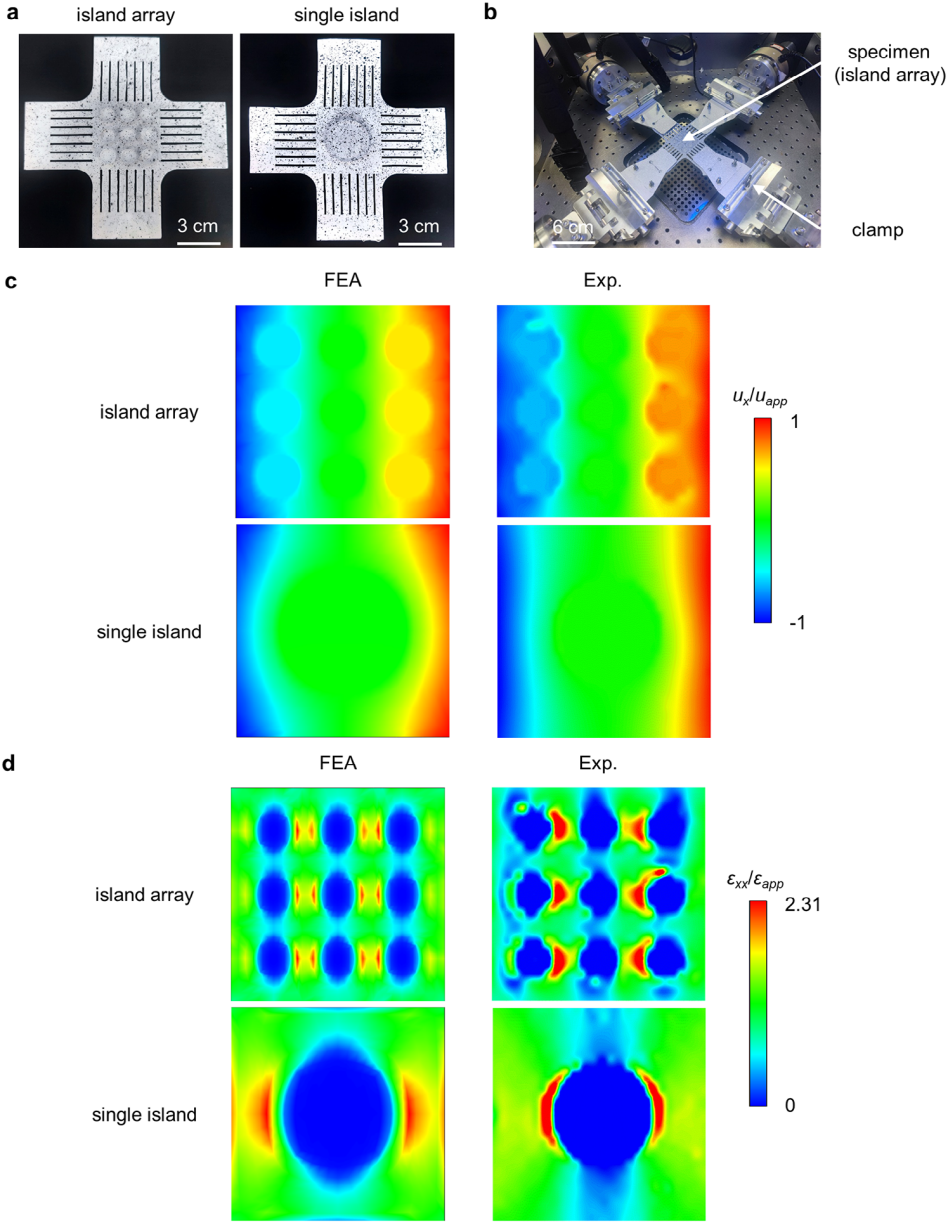
Island effect in array structures. a,b) Optical image of the specimens and the experiment setup. c) Contour plots of *u_x_*/*u_app_* for the island array and single island, based on the results of FEA and experiment. d) Contour plots of *ε_xx_*/*ε_app_* for the island array and single island. Scale bars, 3 cm in a) and 6 cm in b).

Equal biaxial tensile tests are utilized to compare the strain field of the substrate with the 3 × 3 array structure to that with a single island, controlling the equivalent radii the same (**Figure** **8**a,b). The radius of each small island in the array is 0.2*L*, and the interval length is *L*/15. The radius of the single island is 0.6*L*. The area coverage ratios of the two structures are both 0.283.

Figure 8c presents the contour plots of the normalized displacement component along the *x*‐direction (*u_x_*/*u_app_*) on the substrate surface. In practical conditions, the displacement field can be influenced by the size of rigid islands, even when maintaining a constant area coverage ratio *η*. On the one hand, the outer islands of the array exhibit a non‐zero rigid body displacement caused by the deviation from the geometric center of the substrate. On the other hand, the boundary effect of the island array with small size is different from that of a large single island according to Saint‐Venant's principle.

Figure 8d presents the distribution of the normalized normal strain component along the *x*‐direction (*ε_xx_*/*ε_app_*). The maximum values of normalized strain in the array and the single island are both 2.31, indicating that the area coverage ratio controls the degree of strain concentration. Meanwhile, the strain field surrounding the central island in the array is similar to that of the single island, while the strain concentration region near the outer island is asymmetrical due to boundary effects. Similarly, the experimental results within the region of strain concentration are not accurate enough due to the limited resolution of the DIC device. Besides, due to the heavy and densely arranged island array, the central testing region of the flexible substrate sinks beyond the camera depth of field, which reduces the imaging quality and calculation accuracy.

In summary, influenced by the geometric configuration, the island effect of the periodic array structure shows both commonalities and distinctions compared to the effect of the unit configuration. The scaling law reveals the influence of the coverage ratio *η* on the island effect. The degree of strain concentration between the periodic array structure with multiple small islands and that with fewer large islands may be the same. Therefore, the size of each island can be customized depending on the size of the corresponding functional electronic component for minimization of the stress concentration and maximization of the utilization rate of the surfaces, which is available for the designing and manufacturing of flexible electronic devices.

## 4 Conclusion

In this work, a theoretical mechanical model of the island effect under equal biaxial stretching was established based on the results of simulation and experiments. The basic unit of the periodic array in the flexible electronic device based on the design of island‐bridge structures was extracted, and a corresponding 2D geometric model was established. Quantitative analysis of the displacement distribution influenced by various parameters demonstrates that the normalized island radius is the only critical parameter governing the deformation field under the influence of the island effect. Based on this, the scaling law for the displacement and strain distributions was established by fitting the results of FEA. Furtherly, the criterion differentiating the island effect as a non‐axisymmetric problem or an axisymmetric problem was given. It was observed that the scaling law exhibits good consistency with the analytical solution based on the classical theory when the island is small and the axisymmetric simplification criterion is satisfied. Then, two general examples were presented to illustrate the applicability of the scaling law. The scaling law was further expanded to encompass the application of periodic island arrays by introducing the area coverage ratio. A quantitative interpretation of the deformation mechanisms underlying the island effect could provide theoretical insights for the enhancement of structural safety and deformation capability, thereby facilitating the application of stretchable electronics in critical fields (e.g., biomedical devices^[^ ^73^, ^84^, ^85^, ^86^ ^]^ or wearable electronics,^[^ ^31^, ^81^, ^82^, ^83^ ^]^ etc.) and various scenarios. Note that the theoretical model in this study was based on a significantly simplified representation of the island effect phenomenon. In the future, more generalized geometric configurations, diverse material properties, and loading conditions could be considered for broader application in the field of flexible electronics.

## 5 Experimental Section

### Finite Element Analysis

ABAQUS/Standard v2023 was utilized to solve the deformation field of the elastomer substrate with on‐top rigid islands under equal biaxial stretching. The finite element meshes for the substrate and island were eight‐node solid elements with reduced integration and enhanced hourglass control (C3D8R). Refined meshes guarantee computational precision. The substrate adopts a linear elastic constitutive model, with material parameters defined as *E_s_* = 0.1 MPa and *ν_s_* = 0.49 for large deformation. The island was tied onto the substrate by predefined constraints. During the FEA modeling process, tie constraint in the interaction module of ABAQUS was implemented to simulate the bonding behavior. Specifically, the upper surface of the substrate was designated as the main surface, while the bottom surface of the island was designated as the secondary surface. By utilizing the hyperelastic constitutive model and optimizing the mesh, the simulation method could be improved while the enhancement of the accuracy is slight (Figures S3 and S12, Supporting Information). The constitutive model of the island was linear elastic. Except in **Section** **2.2.1** about the impact of Young's modulus ratio, the material parameters of the island are maintained at *E_i_* = 90 GPa and *ν_i_* = 0.3. This parameter scheme allows FEA analyses to approximate the materials in actual equal biaxial tests and represent typical flexible electronics scenarios, ensuring the authenticity and applicability of the simulation results and the validity of the experimental data. Considering the deformation contours could be calculated and transformed between the deformed and undeformed configurations, all simulation contours are plotted on the undeformed configurations (Figure S13, Supporting Information), which could still accurately characterize the normalized deformation pattern of the island effect. Thus, the experimental results were also calculated and plotted on the undeformed configuration, for validating the theoretical and FEA results.

### Fabrication of the Elastomer Substrate and the Rigid Island

The cross‐shaped specimen in Figure 6 is fabricated from a customized high‐strength isotropic textile material. This material exhibits an elevated elastic modulus (≈8 GPa) and a relatively low elongation rate (< 5%), making it well‐suited for the experiments of the substrate with small deformation. For the preparation of the elastomer substrate with large deformation (Figure 7), silica gel mixed by 1A:1B was poured into the prepared plastic culture dish and cured after 8 h (thickness ≈ 1 mm; Ecoflex 0050A/B, Smooth‐on, USA). The Young's modulus of the silicone was ≈0.1 MPa, and the Poisson's ratio was ≈0.49. Note that slits and fillets were introduced on the arms of all the specimens to enhance the uniformity of strain distribution in the central test region.^[^ ^87^ ^]^ Subsequently, the substrate could be precisely cut into the desired pattern using a laser cutting device (VLS 3.60 DT, Universal Laser Systems, USA). All rigid islands in the experiments are circular glass pieces cut from glass plates.^[^ ^88^ ^]^ The transparency of the glass enables comprehensive monitoring of the deformation across the entire substrate surface, including regions covered by the rigid island. The elastic modulus of the glass was ≈90 GPa, and the thickness was ≈3 mm.

### Establishment of the Experiment System

The experiment setup employs the in situ biaxial mechanical testing system (Care Measurement & Control, China) with a non‐contact video extensometer and customized clamps. The biaxial testing machine was powered by servo motors, enabling high‐precision equal biaxial displacement loading with a stroke of 100 mm and a displacement accuracy of 1 µm. High‐resolution, wide‐field, non‐contact video extensometer captures optical images of the deformation, and the displacement and strain fields were visualized through digital image correlation (DIC, SYMTOP, China). For the small deformation experiment, the applied strain (*ε_app_*) was specified as 2% and the loading rate was 10^−4^ s^−1^. For the large deformation experiment and the periodic island array tensile test, *ε_app_* was specified as 20% and the loading rate was 10^−3^ s^−1^. Here, precise strain‐controlled equal biaxial stretching was achieved through the deformation monitoring capability of the video extensometer, which was capable of collecting real‐time strain data of the preselected marker points and providing feedback for the control of the mechanical testing system.

## Conflict of Interest

The authors declare no conflict of interest.

## Supporting information



Supporting Information

Supplemental Movie 1

## Data Availability

The data that support the findings of this study are available from the corresponding author upon reasonable request.
