# Supplementary material for: Theoretical Model of the Island Effect in Flexible Electronics under Equal Biaxial Stretching
Source: Small. 2025 Apr 7;21(19):2409632. doi: 10.1002/smll.202409632 (PMC12067164; doi:10.1002/smll.202409632)
Supplement: Supplementary file 1 — Supporting Information [file SMLL-21-2409632-s001.docx]

Supporting Information

**Title:** **Theoretical model of the island effect in flexible electronics under equal biaxial stretching**

Zifei Ma, Tao Li, Xiaoyong Liu, Jun Wu*, Haibo Luo*, Yanchu Yang*

Contents

Note S1

Figures S1 to S13

Captions for Movie S1

SI References

**Other supporting information for this manuscript**

Movie S1

Supporting Information Note S1: Displacement and strain distribution of the island-substrate system with small island based on classical mechanical theory

According to the 2D geometric model of the island-substrate cell (**Figure 1**c), the displacement boundary conditions for the inner and outer sides are given by

$\left\{ \begin{matrix} \left. u_{r} \right|_{r=R}=0, \\ \left. u_{\theta} \right|_{r=R}=0, \end{matrix} \right.$ (S1)

$\left\{ \begin{matrix} \left. u_{x} \right|_{x=L}=u_{app}, \\ \left. u_{y} \right|_{y=L}=u_{app}, \\ \left. u_{x} \right|_{x=-L}={-u}_{app}, \\ \left. u_{y} \right|_{y=-L}={-u}_{app}. \end{matrix} \right.$ (S2)

According to the FEA results of the displacement field (**Figure 2**j and k), the distribution of radial displacement (*u_r_*) is assumed to resemble the cosine function (cos4*θ*), and the circumferential displacement (*u_θ_*) demonstrates a combination of a constant term and the sine function (sin4*θ*). Therefore, the stress function could take the following form:

$\phi=f\left( r \right)\cos4\theta+g\left( r \right),$ (S3)

where *f* and *g* are expressions that are only dependent on the radial coordinate *r*. The harmonic equation based on the compatibility equation can be given by

$\nabla^{2}\nabla^{2}\phi=0.$ (S4)

Substituting the stress function into Equation (S4), the above equation can be rewritten as

$\left( \frac{\partial^{2}}{\partial r^{2}}+\frac{1}{r}\frac{\partial}{\partial r} \right)\left( \frac{\partial^{2}}{\partial r^{2}}+\frac{1}{r}\frac{\partial}{\partial r} \right)g+\left( \frac{\partial^{2}}{\partial r^{2}}+\frac{1}{r}\frac{\partial}{\partial r}-\frac{16}{r^{2}} \right)\left( \frac{\partial^{2}}{\partial r^{2}}+\frac{1}{r}\frac{\partial}{\partial r}-\frac{16}{r^{2}} \right)f\cos4\theta=0$ .(S5)

When the radius of the island (*R*) is much smaller than that of the substrate (*L*), the deformation field is approximately axisymmetric (See Section 2.3.1, Axisymmetric simplification criterion for the scaling law), and the influence of the angular coordinate *θ* can be neglected. Thereby, Equation (S5) can be simplified as

$\left( \frac{\partial^{2}}{\partial r^{2}}+\frac{1}{r}\frac{\partial}{\partial r} \right)\left( \frac{\partial^{2}}{\partial r^{2}}+\frac{1}{r}\frac{\partial}{\partial r} \right)g=0.$ (S6)

The corresponding characteristic equation is

$\left[ \left( k-2 \right)\left( k-3 \right)+\left( k-2 \right) \right]\left[ k\left( k-1 \right)+k \right]=0.$ (S7)

The above equation can be simplified as

$\left( k-2 \right)^{2}k^{2}=0.$ (S8)

Consequently, the general solution of function *g* is given by

$g=A\ln r+Br^{2}\ln r+Cr^{2}+D.$ (S9)

Meanwhile, the components of the stress tensor can be given by

$\sigma_{rr}=\frac{1}{r}\frac{\partial g}{\partial r},$ (S10)

$\sigma_{\theta\theta}=\frac{\partial^{2}g}{\partial r^{2}},$ (S11)

$\sigma_{r\theta}=0.$ (S12)

For the isotropic linear elastic materials with small deformation, the constitutive model can be given by

$\varepsilon_{rr}=\frac{1}{E}\left[ \sigma_{rr}-\nu\sigma_{\theta\theta} \right],$ (S13)

$\varepsilon_{\theta\theta}=\frac{1}{E}\left[ \sigma_{\theta\theta}-\nu\sigma_{rr} \right],$ (S14)

$\varepsilon_{r\theta}=\frac{1}{G}\sigma_{r\theta}.$ (S15)

Here, *E* denotes the elastic modulus, *ν* denotes Poisson's ratio, and *G* denotes the shear modulus. Substituting Equation (S9) into Equation (S10)-(S15), the components of the strain tensor can be given by

$\varepsilon_{rr}=\frac{1}{E}\left[ \left( 1+\nu\right)\frac{A}{r^{2}}+\left( 1-3\nu\right)B+2\left( 1-\nu\right)B\ln r+2\left( 1-\nu\right)C \right]$ (S16)

$\varepsilon_{\theta\theta}=\frac{1}{E}\left[ -\left( 1+\nu\right)\frac{A}{r^{2}}+\left( 3-\nu\right)B+2\left( 1-\nu\right)B\ln r+2\left( 1-\nu\right)C \right]$ (S17)

$\varepsilon_{r\theta}\equiv0$ (S18)

Then, the geometric equations of the island-substrate cell can be given by

$\varepsilon_{rr}=\frac{\partial u_{r}}{\partial r}$ (S19)

$\varepsilon_{\theta\theta}=\frac{1}{r}\frac{\partial u_{\theta}}{\partial\theta}+\frac{u_{r}}{r}$ (S20)

$\varepsilon_{r\theta}=\frac{1}{r}\frac{\partial u_{r}}{\partial\theta}+\frac{\partial u_{\theta}}{\partial\theta}-\frac{u_{\theta}}{r}$ (S21)

Substituting Equation (S16)-(S18) into Equation (S19)-(S21), displacement distribution can be obtained by integrating, given as

$u_{r}=\frac{1}{E}\left[ -\left( 1+\nu\right)\frac{A}{r}+\left( 1-3\nu\right)Br+2\left( 1-\nu\right)Br\left( \ln r-1 \right)+2\left( 1-\nu\right)Cr \right]+I\cos\theta+K\sin\theta,$ (S22)

$u_{\theta}=\frac{4Br\theta}{E}-\int\left( I\cos\theta+K\sin\theta\right) \text{d}\theta+Hr+F,$ (S23)

where *A*, *B*, *C*, *I*, *K*, *H*, and *F* are undetermined coefficients. The coefficients *I* and *K* are introduced to describe the rigid body rotation, and *H* and *F* are introduced to describe the rigid body displacement. Considering the island-substrate system without rigid body rotation and displacement, these four coefficients equal zero. In addition, since *u_θ_* denotes the same value when *θ* equals 0 or 2π, the coefficient *B* equals zero. Hence, Equation (S22) and (S23) can be further simplified as

$u_{r}=\frac{1}{E}\left[ -\left( 1+\nu\right)\frac{A}{r}+2\left( 1-\nu\right)Cr \right],$  (S24)

$u_{\theta}\equiv0.$ (S25)

When *r* is equal to *R*, the inner boundary condition specified by Equation (S1) is substituted, and the coefficient *A* can be solved as

$A=\frac{2C\left( 1-\nu\right)}{1+\nu}R^{2}.$ (S26)

When *θ* is equal to 0 and *r* is equal to *x* and *L*， the outer boundary condition equation should be satisfied and the displacement *u_r_* is equal to *u_x_* and *u_app_*. By substituting Equation (S2) into Equation (S24), the coefficient *C* can be solved as

$C=\frac{Eu_{app}}{2\left( 1-\nu\right)}\cdot\frac{L}{L^{2}-R^{2}}.$ (S27)

Substituting Equation (S26) and (S27) into Equation (S24), the displacement distribution of the island-substrate system with a small island can be given by

$\frac{u_{r}}{u_{app}}=\left\{ \begin{matrix} 0, & r\leq R \\ \frac{L}{r}\left( \frac{r^{2}-R^{2}}{L^{2}-R^{2}} \right), & r>R \end{matrix} \right.,$ (S28)

$\frac{u_{\theta}}{u_{app}}\equiv0.$ (S29)

Finally, through the differential method, the strain distribution can be given by

$\frac{\varepsilon_{rr}}{\varepsilon_{app}}=\left\{ \begin{matrix} 0, & r\leq R \\ \frac{L^{2}\left( r^{2}+R^{2} \right)}{r^{2}\left( L^{2}-R^{2} \right)}, & r>R \end{matrix} \right.,$ (S30)

$\frac{\varepsilon_{\theta\theta}}{\varepsilon_{app}}=\left\{ \begin{matrix} 0, & r\leq R \\ \frac{L^{2}\left( r^{2}-R^{2} \right)}{r^{2}\left( L^{2}-R^{2} \right)}, & r>R \end{matrix} \right.,$ (S31)

$\frac{\varepsilon_{r\theta}}{\varepsilon_{app}}=0.$ (S32)


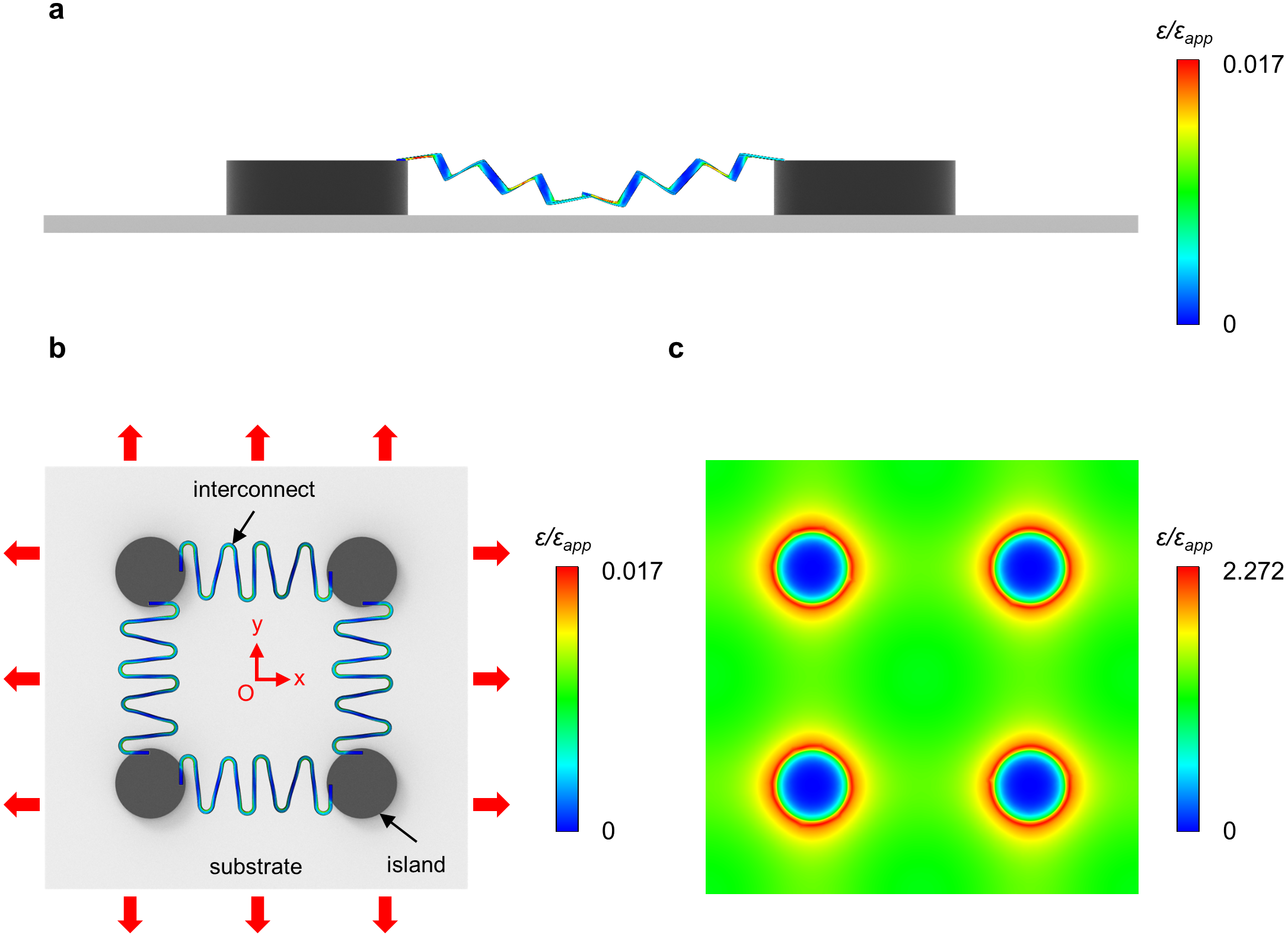


**Figure S1.** FEA results of a stretched island-bridge structure. a) Front view and b) top view of the normalized maximum principal strain distribution (*ε/ε_app_*) in the interconnects. c) Top view of the *ε/ε_app_* contour on the substrate surface. *ε_app_* is the applied strain value.


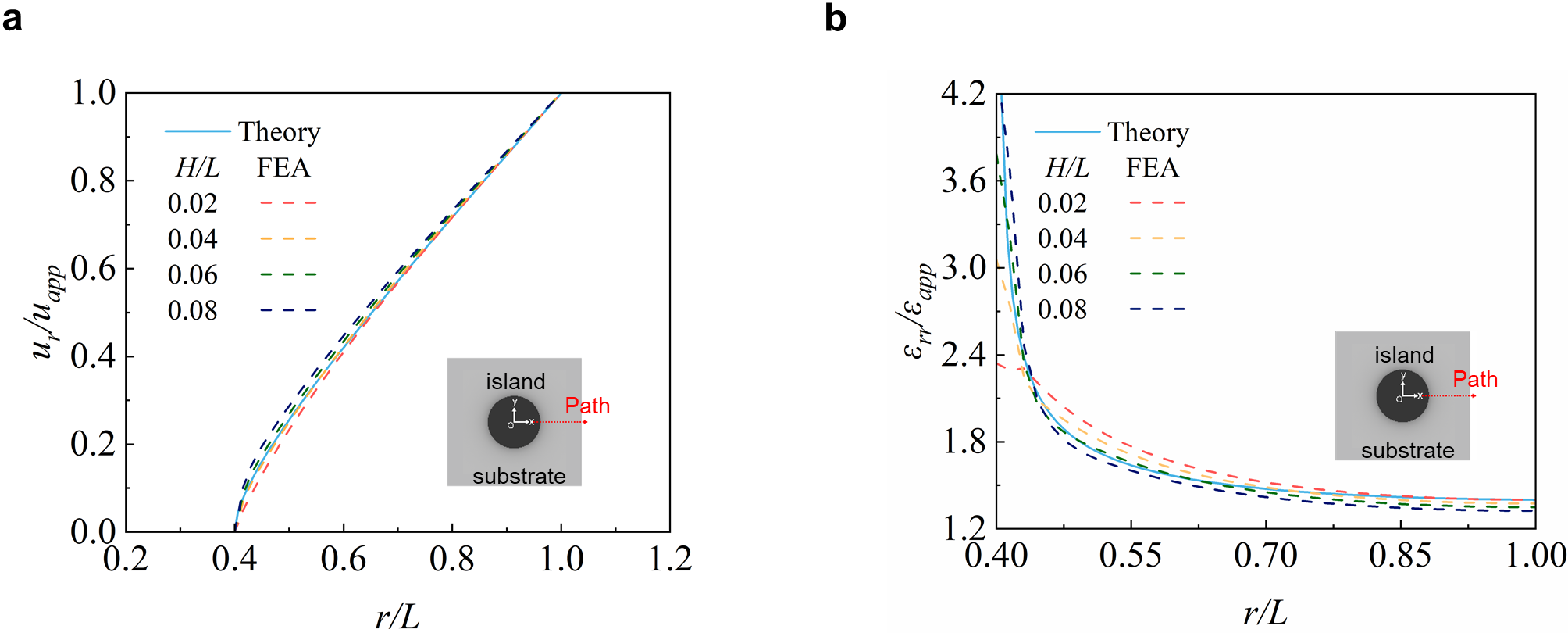


**Figure S2.** Effect of the normalized substrate thickness (*H*/*L*) on displacement and strain distribution. a) Distribution of normalized radial displacement (*u_r_*/*u_app_*) along radial direction (*θ*=0) at the substrate surface with different levels of *H*/*L*. b) Distribution of ε*_rr_*/*ε_app_* along the radial direction under different substrate thickness.


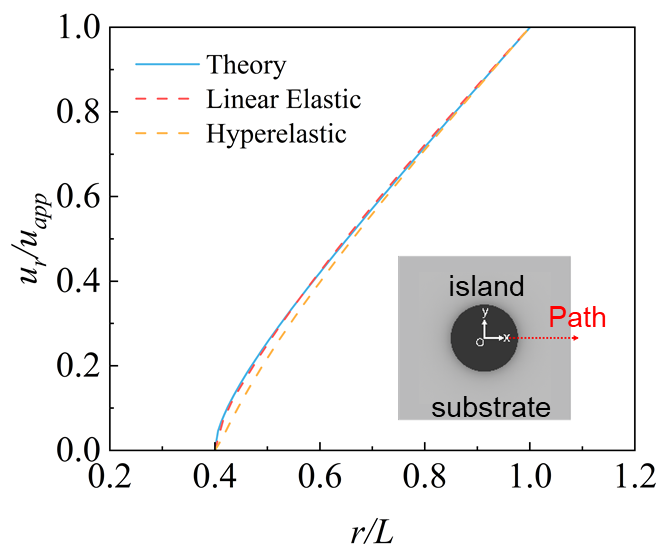


**Figure S3.** Comparison between different constitutive models of substrate material. Distribution of normalized radial displacement (*u_r_*/*u_app_*) along radial direction (*θ*=0) at the substrate surface by utilizing different constitutive models (linear elasticity and hyperelasticity) of the substrate model.


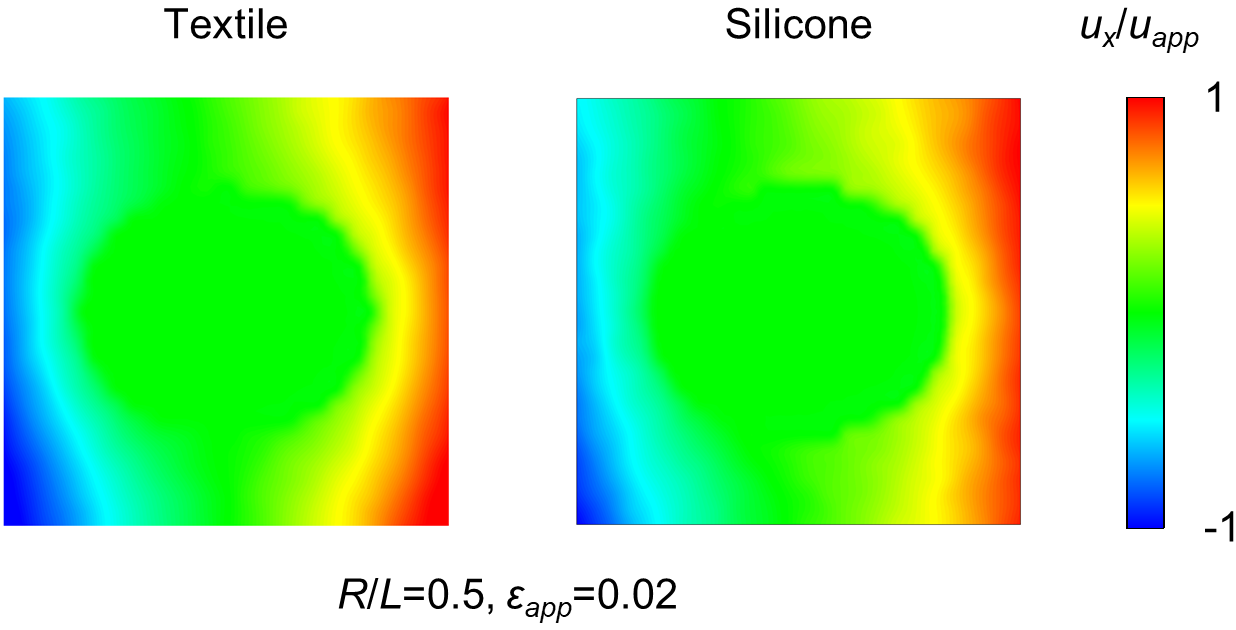


**Figure S4.** Experimental contour plots of normalized displacement along the *x* direction in the central test zone of the textile specimen (left) and the silicone specimen (right) under the equal biaxial stretching (*ε_app_*≈2%).


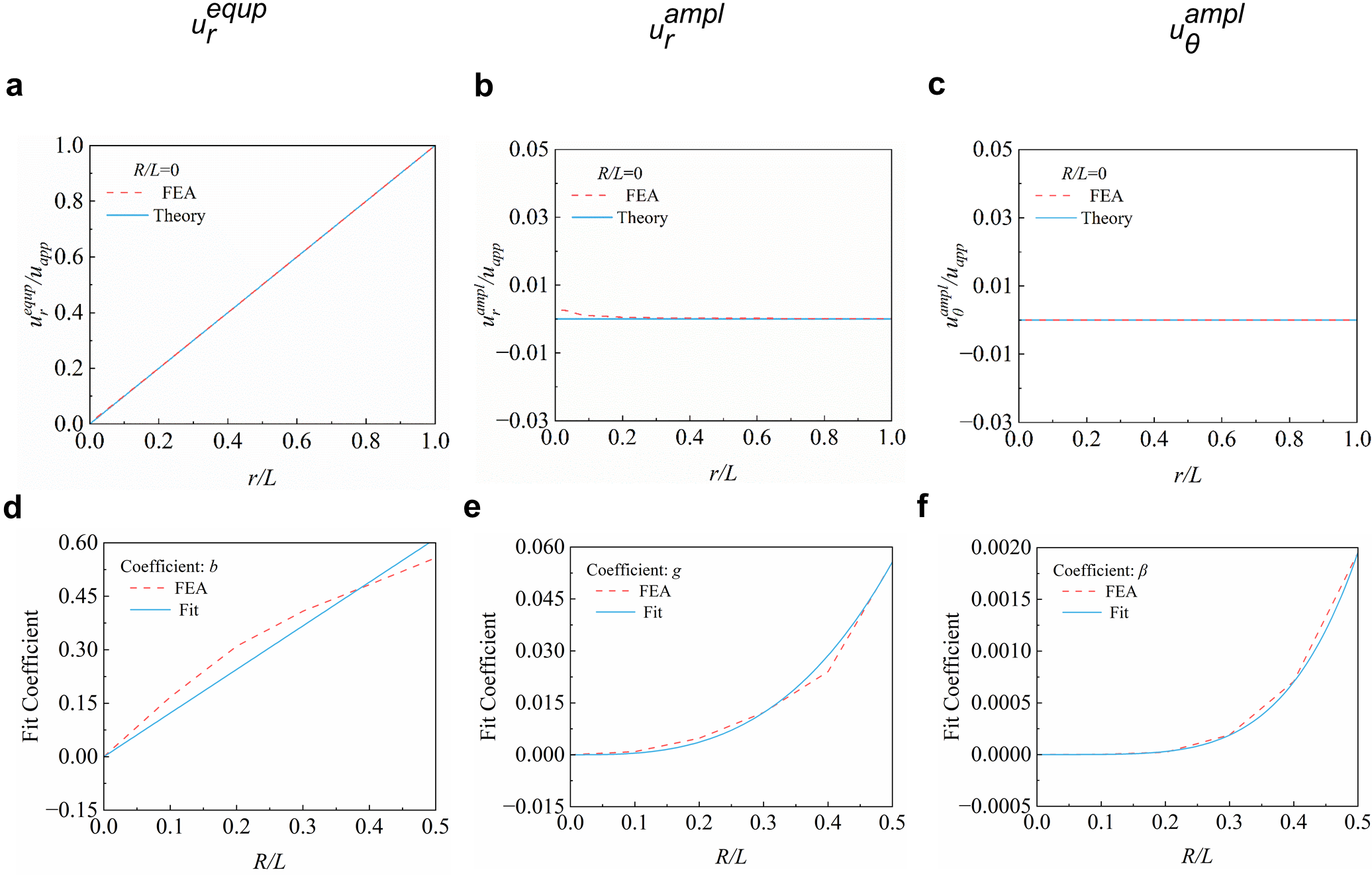


**Figure S5.** Fitting of the expressions for the displacement characteristic quantities. Distribution of a) *uequp r*/*u_app_*, b) *uampl r*/*u_app_*, and c) *uampl θ*/*u_app_* along radial direction (*θ*=0) for *R*/*L*=0. Fitting of the coefficients: *d*) *b*, e) *g*, and f) *β*.


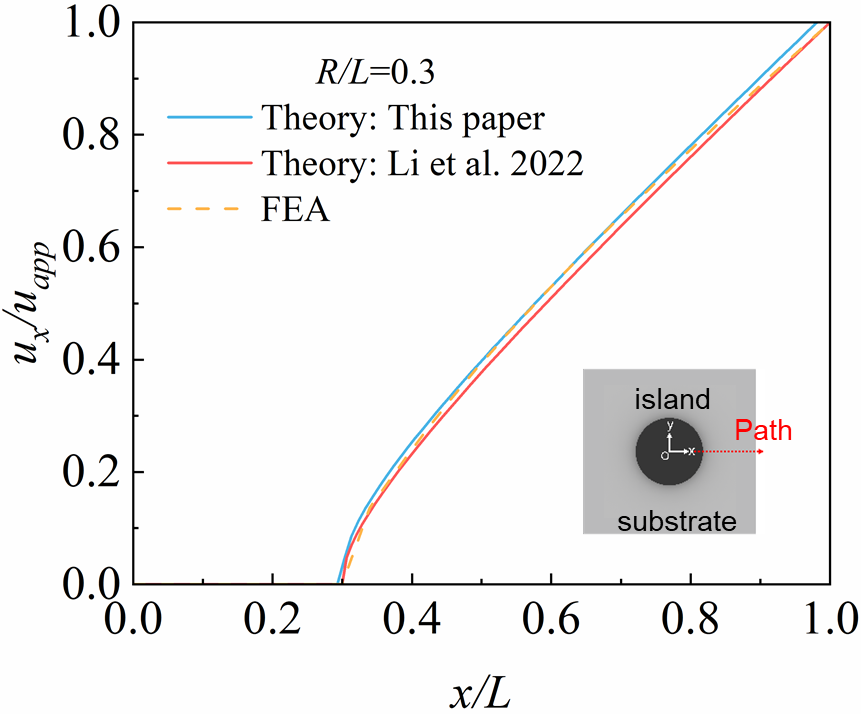


**Figure S6.** Comparison of displacement (*u_r_*/*u_app_*) curves based on theoretical^[1]^ and FEA results.


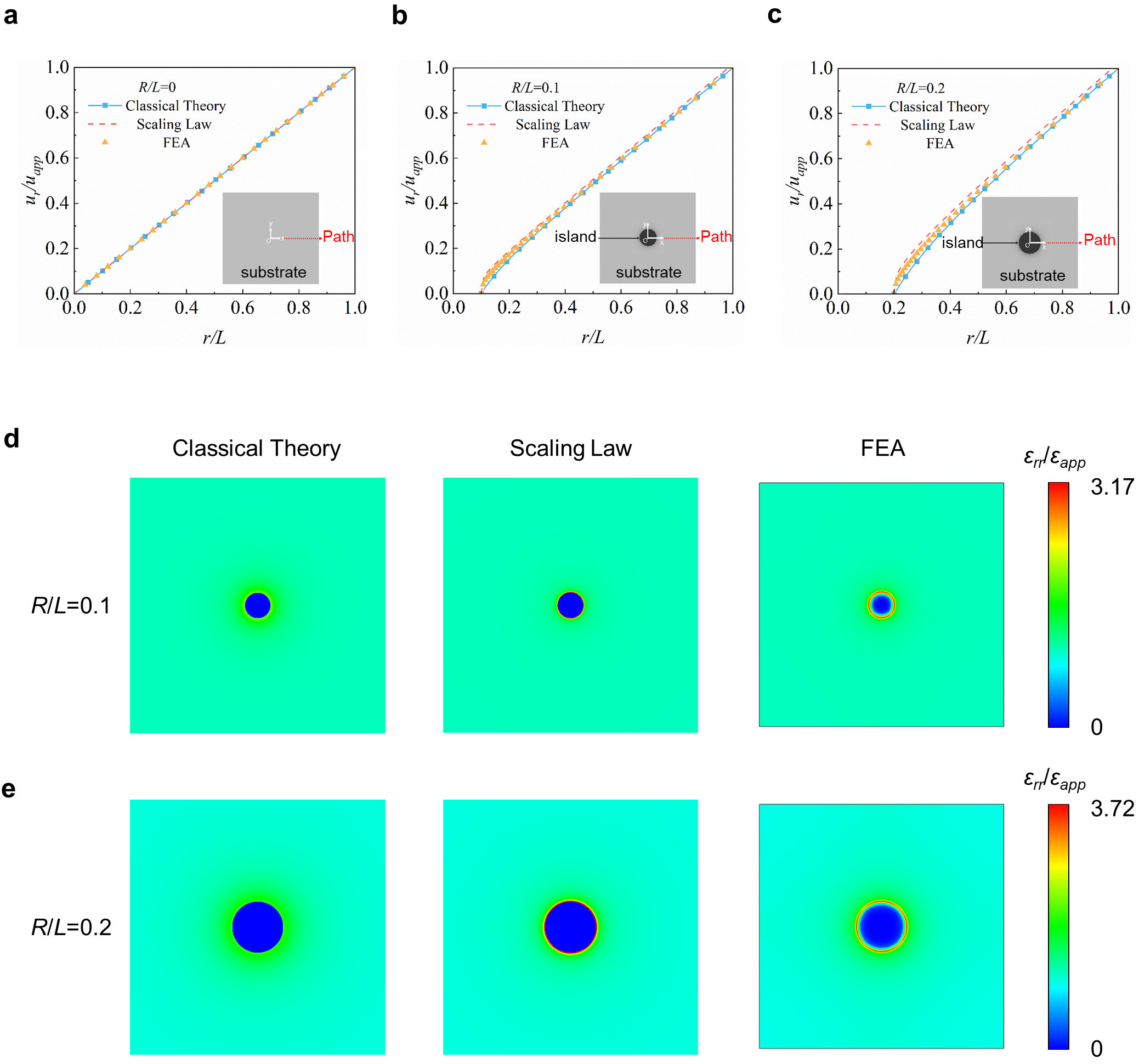


**Figure S7.** Comparison of Scaling Laws with Classical Theory. a) Distribution of *u_r_*/*u_app_* along radial direction (*θ*=0) for *R*/*L*=0. b) Distribution of *u_r_*/*u_app_* for *R*/*L*=0.1. c) Distribution of *u_r_*/*u_app_* for *R*/*L*=0.2. Contour plots of ε*_rr_*/*ε_app_* based on classical theory, scaling law and FEA results, with normalized island radius *R*/*L*= d) 0.1 and e) 0.2.


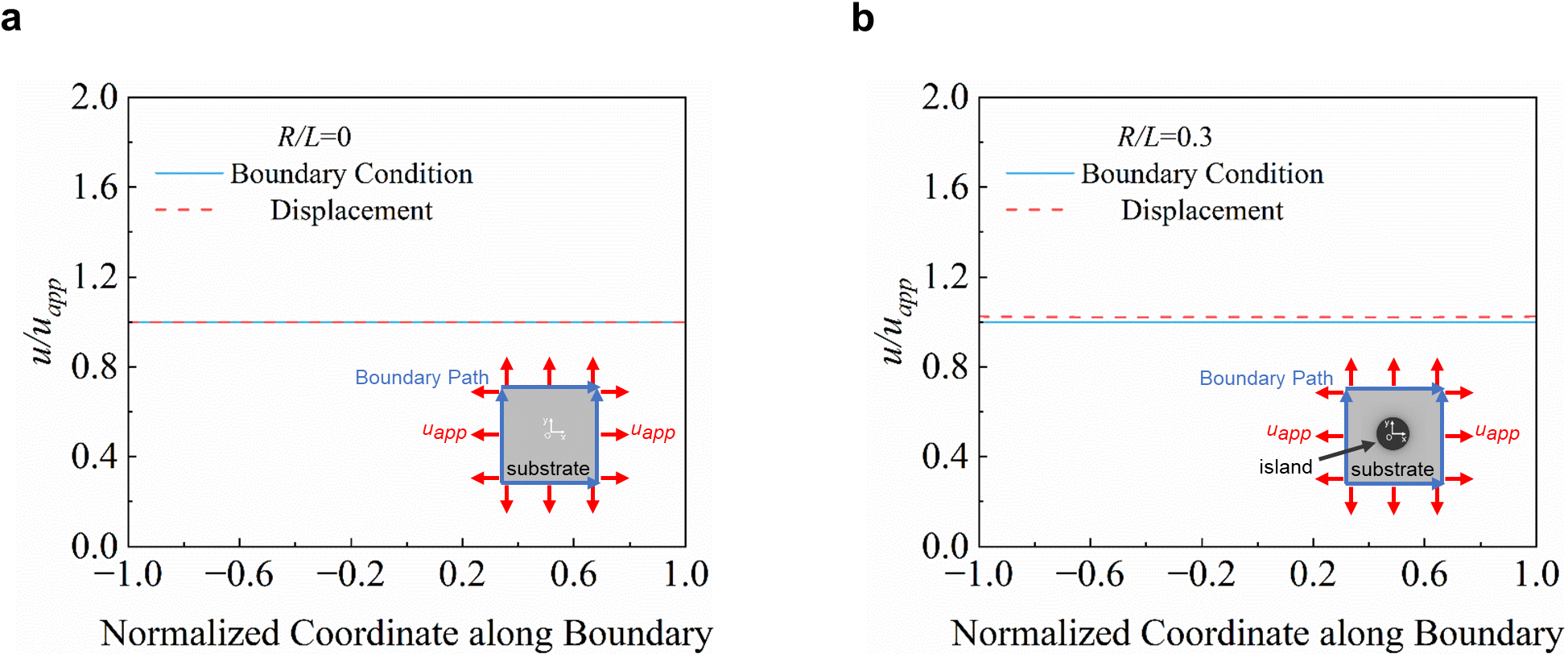


**Figure S8.** Verification of ideal outer boundary conditions. Comparison of the displacement distribution at outer unit boundaries with ideal boundary conditions when *R*/*L* = a) 0, b) 0.3.


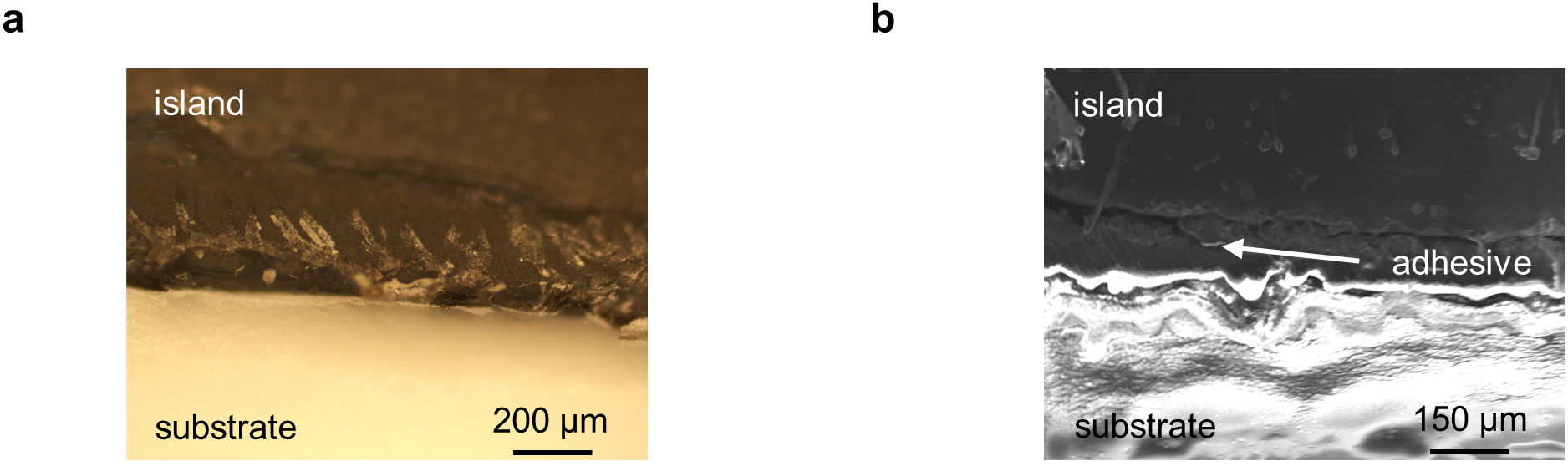


**Figure S9.** a) Optical microscope image (Scale bar, 200μm) and b) scanning electron microscope image (Scale bar, 150μm) of the bonding interface in a stretched rigid-island/soft-substrate specimen.


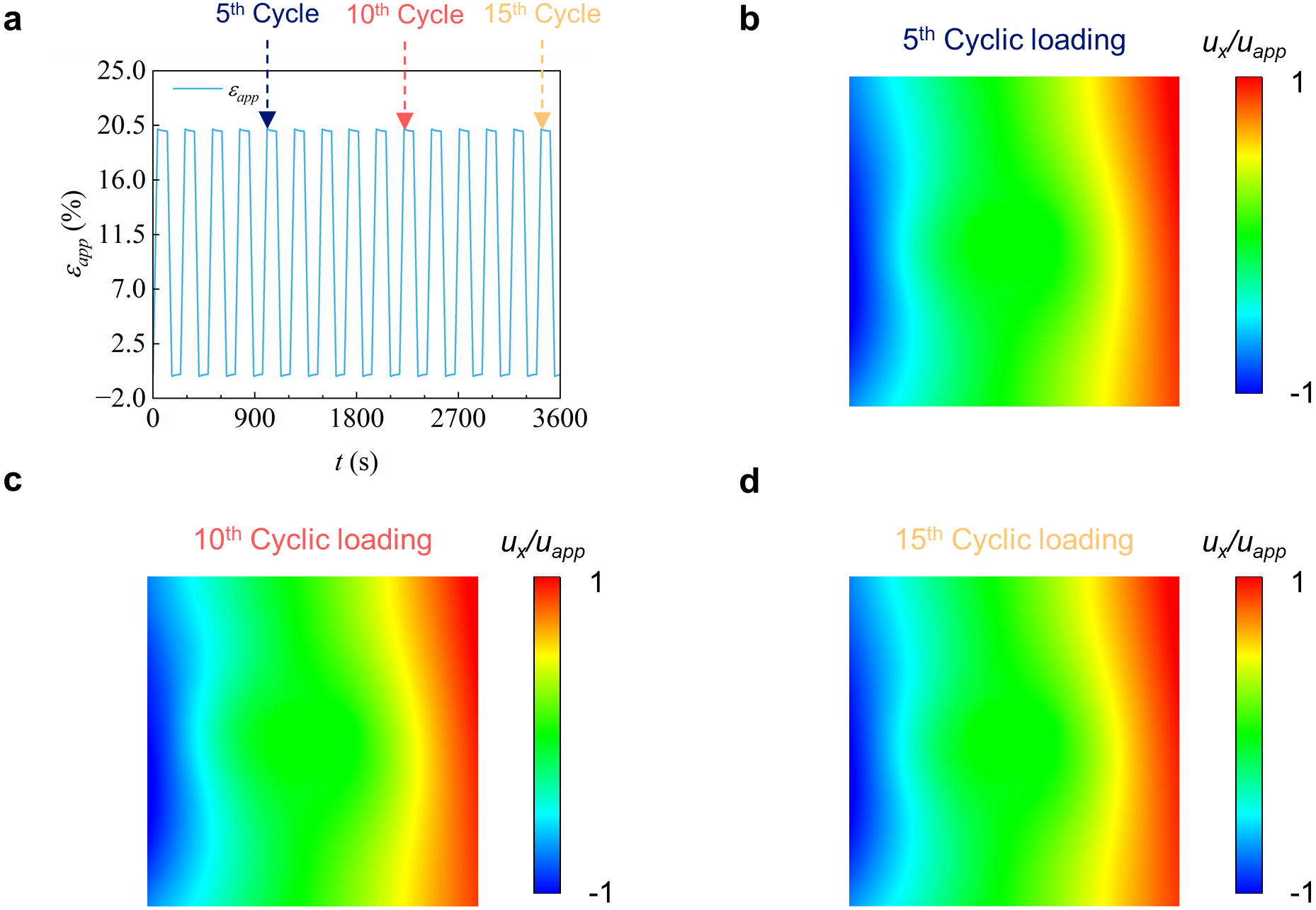


**Figure S10.** Cyclic tensile test of the island-substrate structure. a) Load-time relation curve, with arrows indicating the cycles of interest. Normalized *x*-directional displacement contours after tensile loading in the b) 5^th^, c) 10^th^ and d) 15^th^ cycle.


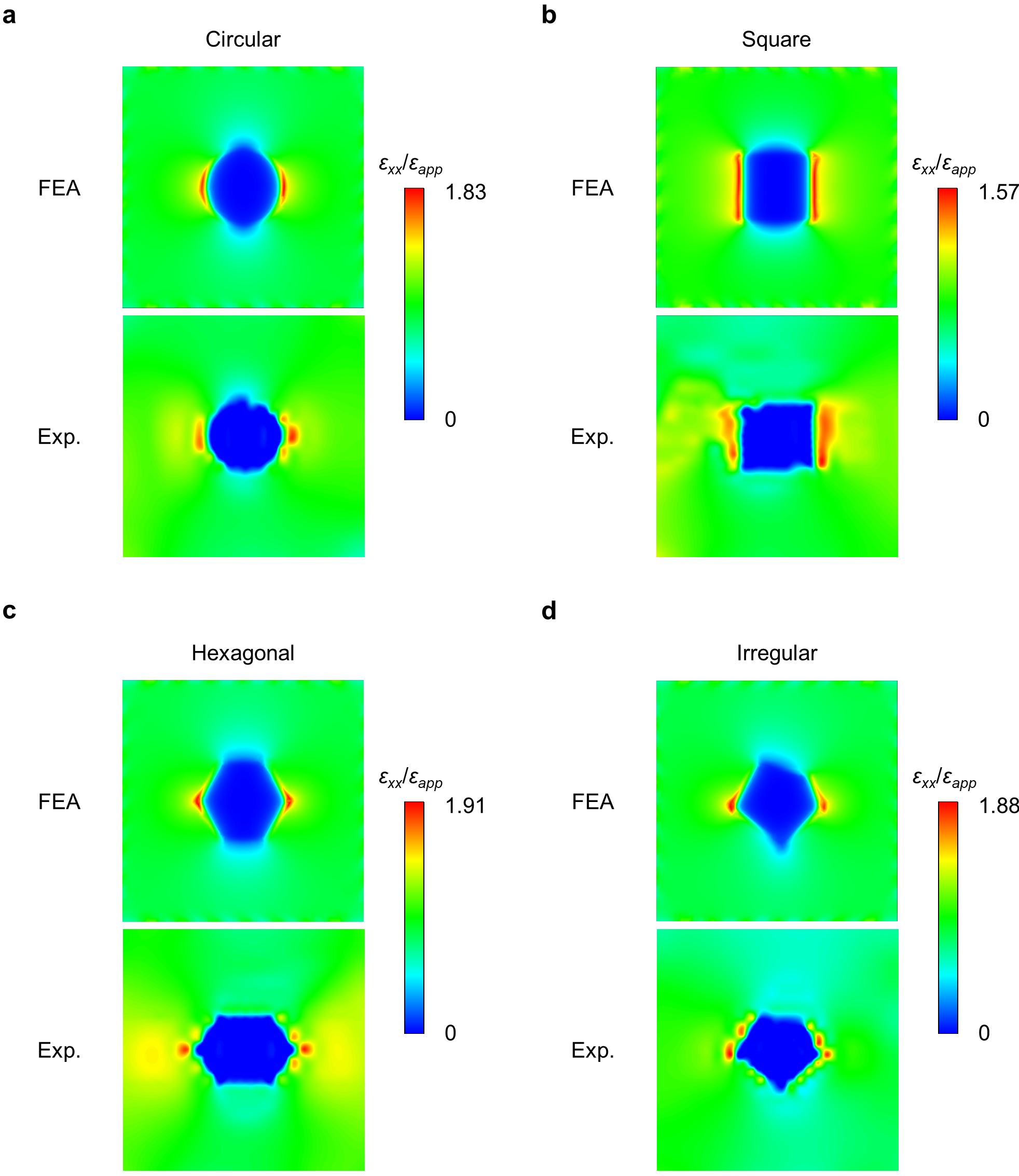


**Figure S11.** Influence of rigid island geometry on substrate strain concentration. FEA and experimental normalized *x*-directional normal strain contours of substrates with a) circular, b) square, c) hexagonal, and d) irregular pentagonal island. Note that the areas of these islands are designed to be approximately equal.


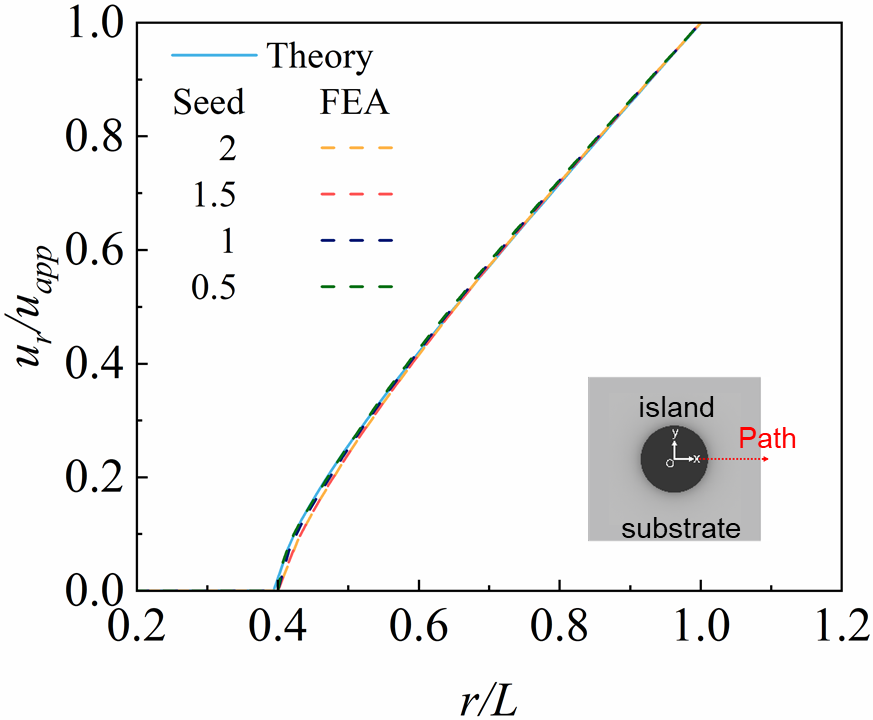


**Figure S12.** Distribution of normalized radial displacement (*u_r_*/*u_app_*) along radial direction (*θ*=0) at the substrate surface for different mesh seed sizes.


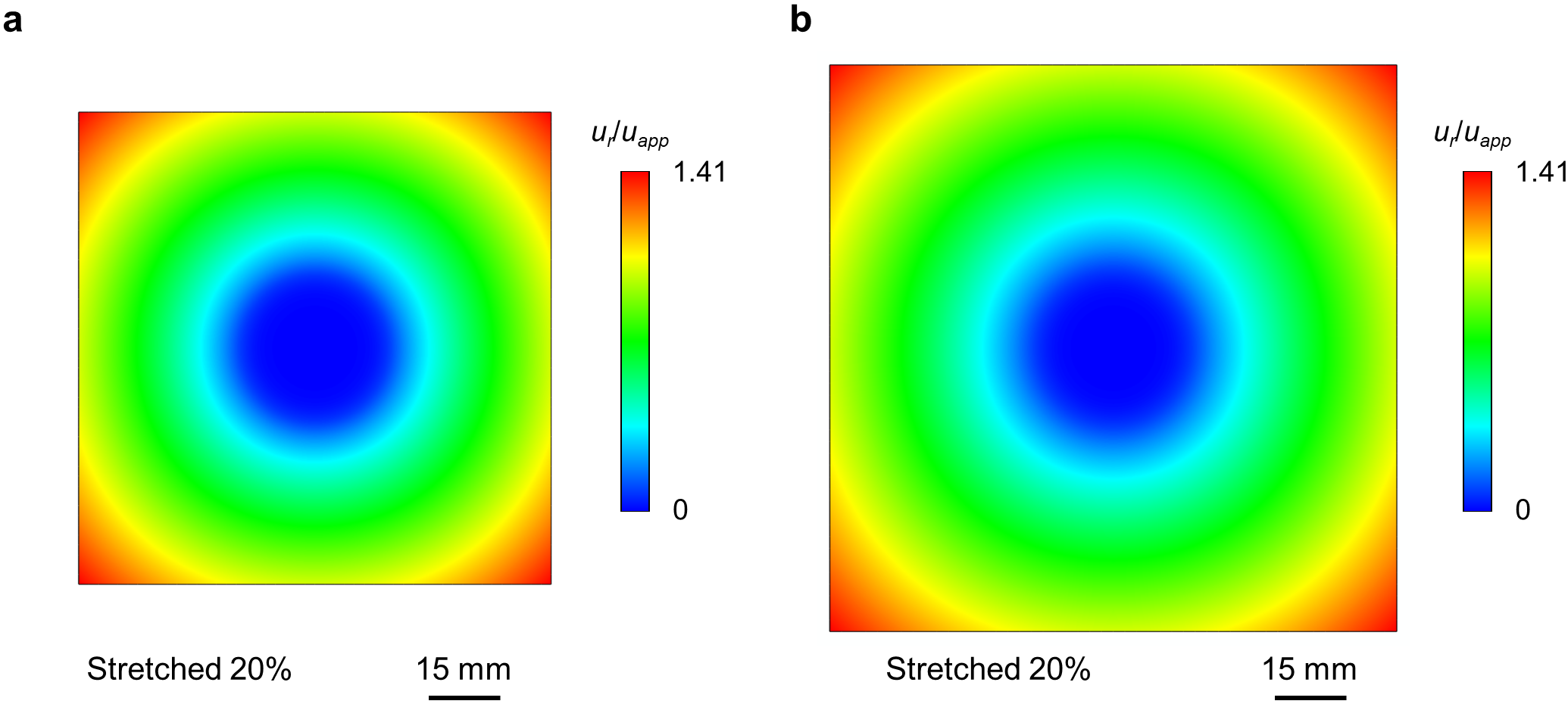


**Figure S13.** FEA contours of *u_r_*/*u_app_* plotted on a) undeformed and b) deformed shape. Scale bars, 15 mm.

**Movie S1 (separate file).** Video record of the cyclic loading test process.

**SI References**

[1] K. Li, Y. Shuai, X. Cheng, H. Luan, S. Liu, C. Yang, Z. Xue, Y. Huang, Y. Zhang, *Small* **2022**, *18*, 2107879.
